# Molecular Recognition of Natural and Non‐Natural Substrates by Cellodextrin Phosphorylase from *Ruminiclostridium Thermocellum* Investigated by NMR Spectroscopy

**DOI:** 10.1002/chem.202102039

**Published:** 2021-10-08

**Authors:** Valeria Gabrielli, Juan C. Muñoz‐García, Giulia Pergolizzi, Peterson de Andrade, Yaroslav Z. Khimyak, Robert A. Field, Jesús Angulo

**Affiliations:** ^1^ School of Pharmacy University of East Anglia Norwich Research Park Norwich NR4 7TJ UK; ^2^ Department of Biological Chemistry John Innes Centre Norwich Research Park Norwich NR4 7TH UK; ^3^ Present address Department of Chemistry and Manchester Institute of Biotechnology University of Manchester Manchester M1 7DN UK; ^4^ Department of Organic Chemistry Faculty of Chemistry University of Seville 41012 Seville Spain; ^5^ Instituto de Investigaciones Químicas (CSIC-US) Avda. Américo Vespucio, 49 41092 Sevilla Spain

**Keywords:** cellodextrin phosphorylase, ligand-based NMR spectroscopy, molecular docking, protein-ligand interactions

## Abstract

β‐1→4‐Glucan polysaccharides like cellulose, derivatives and analogues, are attracting attention due to their unique physicochemical properties, as ideal candidates for many different applications in biotechnology. Access to these polysaccharides with a high level of purity at scale is still challenging, and eco‐friendly alternatives by using enzymes in vitro are highly desirable. One prominent candidate enzyme is cellodextrin phosphorylase (CDP) from *Ruminiclostridium thermocellum*, which is able to yield cellulose oligomers from short cellodextrins and α‐d‐glucose 1‐phosphate (Glc‐1‐P) as substrates. Remarkably, its broad specificity towards donors and acceptors allows the generation of highly diverse cellulose‐based structures to produce novel materials. However, to fully exploit this CDP broad specificity, a detailed understanding of the molecular recognition of substrates by this enzyme in solution is needed. Herein, we provide a detailed investigation of the molecular recognition of ligands by CDP in solution by saturation transfer difference (STD) NMR spectroscopy, tr‐NOESY and protein‐ligand docking. Our results, discussed in the context of previous reaction kinetics data in the literature, allow a better understanding of the structural basis of the broad binding specificity of this biotechnologically relevant enzyme.

## Graphical abstract

**Understanding** the molecular basis of the broad specificity of cellodextrin phosphorylase (CDP) from *Ruminiclostridium thermocellum*, can facilitate the generation of highly diverse CDP‐catalysed cellulose‐based structures. Here, NMR spectroscopy and molecular modelling allowed to understand how CDP is able to bind non‐natural chemically modified donors and acceptors with different binding epitopes, revealing the structural requirements for CDP ligands, which will enable exploitation of CDP to produce novel materials of high biotechnological interest.

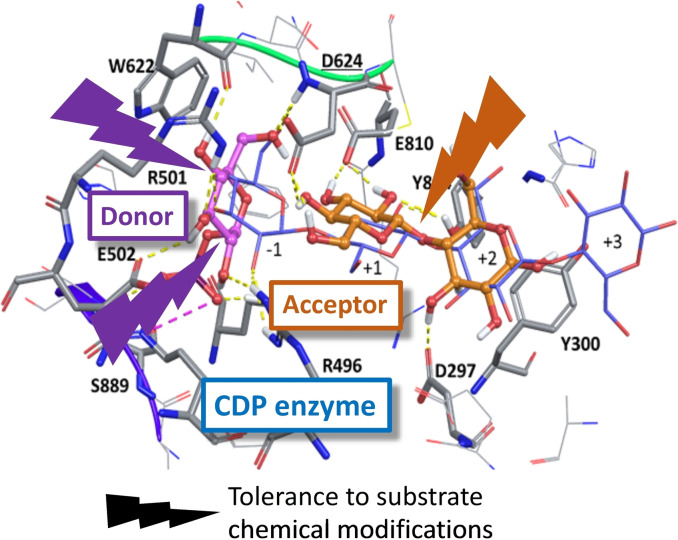

## Introduction

Carbohydrate‐active enzymes (CAZy) are valuable alternative tools to the traditional chemical synthesis of glycans. Among these biocatalysts, glycoside phosphorylases (GPs) have been used to synthesize a broad range of glycosides in a regio‐ and stereo‐specific manner. In particular, cellodextrin phosphorylases (CDP, EC 2.4.1.49) have recently attracted attention for their potential to produce tailor‐made cellulose‐like materials with highly ordered nanostructures, as well as short‐chain soluble oligosaccharides. These materials find application as possible ingredients for animal and human nutrition^[1]^ and for the development of novel all‐cellulose paper‐based devices.^[2]^

CDP belongs to the GH94 glycosyl hydrolase family and was first reported in 1967 by Sheth and Alexander.^[3]^ To date, CDPs have been isolated from several bacterial sources, including *Ruminiclostridium thermocellum,* ^[3]^ *Clostridium stercorarium,* ^[4]^ *Ruminococcus albus,* ^[5]^ *Thermosipho africanus* ^*[6]*^ and *Ruminiclostridium cellulolyticum*.^*[7]*^ A number of GH94 CDPs have been cloned, expressed and characterized,[^4^, ^6^, ^7^, ^8^] with CDP from *Ruminiclostridium thermocellum* and *Clostridium stercorarium* being the most studied.

CDP catalyses the phosphorolysis and reverse phosphorolysis of cellooligosaccharides. In the first of these reactions, the inter‐glycosidic linkage of cellooligosaccharides longer than d‐cellobiose are broken, whereas in the reverse reaction cellooligosaccharides are synthesized using α‐d‐glucose 1‐phosphate (Glc‐1‐P) as donor and d‐cellobiose as acceptor. Broad specificity towards non‐natural substrates was first demonstrated by Samain et al., who pioneered the use of non‐natural substrates in CDP‐catalysed reactions using 4‐thiocellobiose, methyl β‐cellobioside, and methyl 4‐thio‐a‐cellobioside as acceptors, showing the effectiveness of CDP in the synthesis of diverse functionalized oligosaccharides.^[9]^ The relaxed substrate specificity of CDP is illustrated by the number of donor‐ and acceptor‐like ligands known to be recognized by the enzyme (see full list in the Supporting Information Tables S1–S5). Specifically, recent works report the use of β‐d‐glucose acceptors functionalized at the anomeric position with non‐reactive (hydrophilic and hydrophobic) groups or reactive substituents (2‐(glucosyloxy)ethyl methacrylate, thiol, 2‐azidoethyl, etc) to control the self‐assembly process or to provide additional reactivity for post‐synthesis modification, respectively.[^2^, ^10^] Conversely, the use of functionalized donors has been underexploited.

The 3D structure of CDP was elucidated by X‐ray crystallography, in its apo form and bound to the tetrasaccharide d‐cellotetraose (PDB: 5NZ7 apo structure; 5NZ8 ligand bound).^[11]^ The study revealed key interactions for cellotetraose recognition at the catalytic cleft subsites −1, +1, +2 and +3. However, structural details of the molecular recognition of donor and acceptor‐like molecules with DP lower than 4 have yet to be reported, which are necessary to deepen our understanding of the molecular basis of such a broad specificity. For weak binders, such as non‐natural CDP ligands, structural information must be gained under the dynamic conditions existing in solution, which better reflect the rapid ligand binding kinetics which are not observable in the crystalline state employed for X‐ray crystallography.

We have previously used X‐ray crystallography and saturation transfer difference NMR spectroscopy (STD NMR) to investigate substrate recognition with β‐1,3 glucan phosphorylases.^[12]^ Here, we have applied the high‐resolution ligand‐based NMR techniques STD NMR and transferred NOESY (tr‐NOESY) experiments, in combination with molecular modelling calculations, to gain structural information on the interactions of CDP with donor‐ and acceptor‐like ligands. For weak binding ligands, STD NMR is a powerful technique to map the key protons of the ligand for protein interaction (binding epitope mapping),^[13]^ as well as to determine protein‐ligand dissociation constants (K_D_).^[14]^ Further, tr‐NOESY experiments allow the measurement of ligand intramolecular proton‐proton distances in the bound state, reporting on the ligand bioactive conformation.^[15]^

Strikingly, our STD NMR experiments demonstrate for the first time that phosphate anion (co‐substrate in the enzymatic catalysis) plays a role in CDP acceptor binding affinity, while not significantly impacting its binding mode. Our study provides structural information at atomic detail that will inform the rational design of synthetic substrate analogues for CDP with appropriate decorations for the production of novel cellulose‐based materials.

## Results and Discussion

### Structural basis of molecular recognition of natural and non‐natural donor ligands

For this study, we first chose the natural donor substrate Glc‐1‐P and donor‐like molecules, based on previous kinetics studies indicating that some modifications on the hexopyranose ring of the sugar 1‐phosphate ligands have a significant impact on the enzymatic activity of CDP.[^11^, ^16^] Accordingly, besides the CDP natural substrate (Glc‐1‐P), a series of non‐natural sugar 1‐phosphate molecules were selected for investigation, including α‐d‐galactose‐1‐phosphate (Gal‐1‐P) and α‐d‐mannose‐1‐phosphate (Man‐1‐P), as well as functionalised glucose analogues such as α‐d‐glucosamine‐1‐phosphate (GlcN‐1‐P) and 6‐deoxy‐6‐fluoro‐α‐d‐glucose‐1‐phosphate (6F‐Glc‐1‐P).

### Binding detection by STD NMR: Molecular recognition of the natural donor substrate Glc‐1‐P

We first confirmed that, for all the selected ligands, binding to CDP was detectable by STD NMR (Supporting Information Figure S1). Then, in order to gain structural information on the ligand‐enzyme complexes, series of STD NMR experiments at different saturation times were carried out, monitoring the growth of saturation transfer for every proton of the ligands (STD build‐up curves, Supporting Information Figures S2‐S6). From these curves, the corresponding ligand binding epitope mappings were determined using the initial growth rates approach^[17]^ (Supporting Information Tables S6, S8–S10). These mappings result from the positioning of the ligand within the protein binding pocket and report on the binding mode of the ligands, highlighting areas intimately recognized by the protein in the bound state,^[13]^ although they do not reveal the nature of the interactions responsible for the molecular recognition. In this work, the comparison of the binding epitope mappings of different ligands of CDP allows to identify changes in the modes of binding due to modifications in the chemical structures of the ligands. The discussion of these results in terms of previous reaction kinetics data existing in the literature, helps to reach a better understanding of the molecular recognition of ligands by CDP, providing insights into the structural basis of the broad binding specificity that makes this enzyme biotechnologically relevant.

**Figure 1:**
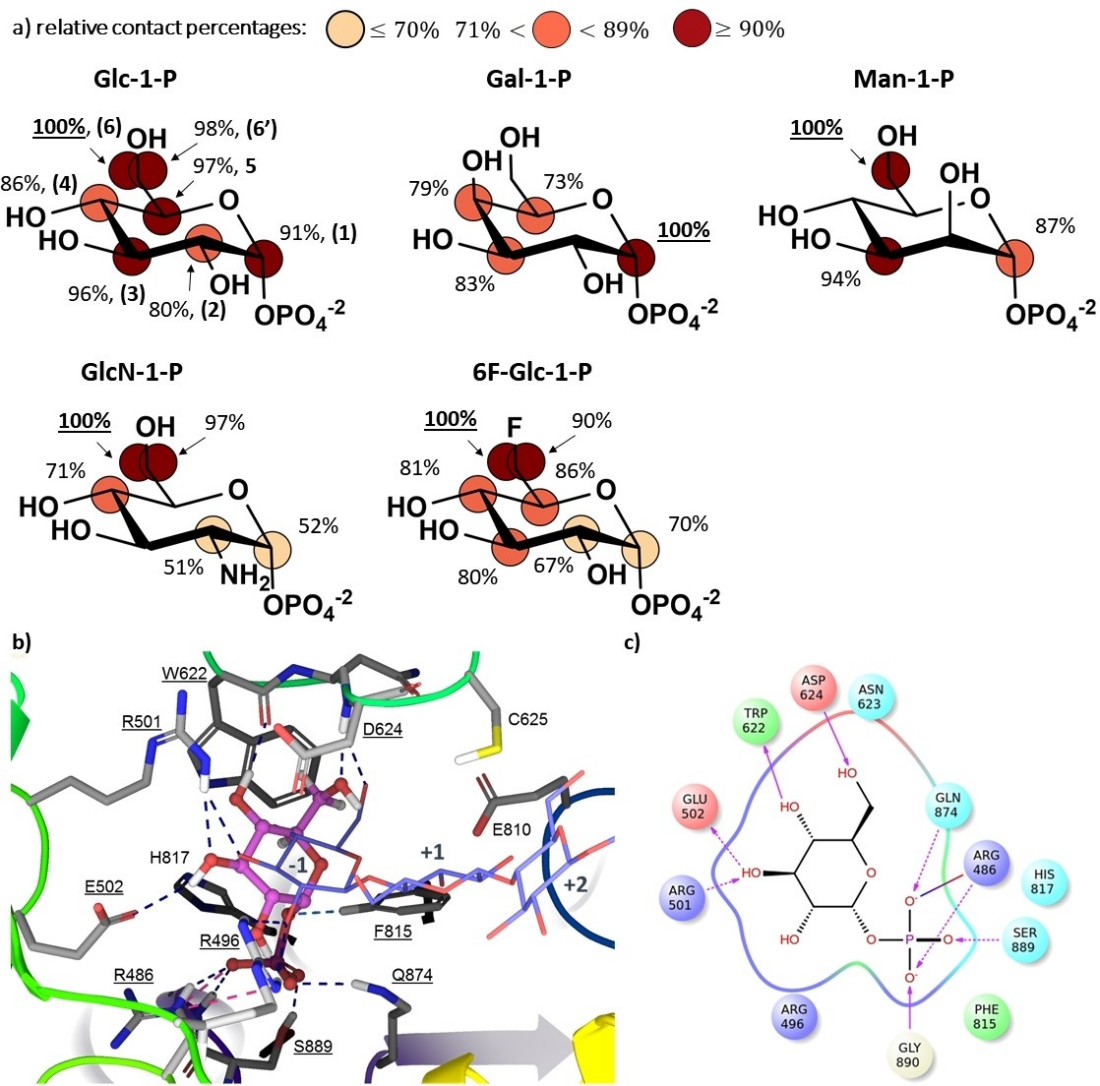
Molecular recognition of natural and non‐natural donor‐like ligands by CDP. a) Binding epitope mappings from STD NMR for the interactions of CDP with the donor substrate Glc‐1‐P as well as with the non‐natural ligands Gal‐1‐P, Man‐1‐P, GlcN‐1‐P and 6F‐Glc‐1‐P. STD NMR experiments were carried out with samples containing 1 : 200 “binding site to ligand ratio” for Glc‐1‐P, and 1 : 100 ratio for Gal‐1‐P, Man‐1‐P, GlcN‐1‐P and 6F‐Glc‐1‐P. All the experiments were carried at 800 MHz and 278 K in [D_11_]Tris 25 mM, pH 7.4 (NaCl 100 mM). Glucopyranose atoms are numbered in Glc‐1‐P (top left). b) 3D docking model of the CDP/Glc‐1‐P complex. The cellotetraose complex (PDB ID 5NZ8, purple wire representation)^[11]^ is superimposed for comparison. For simplicity, only three rings of cellotetraose are shown (sites −1, +1 and +2). Glc‐1‐P is represented in pink ball‐and‐sticks and interacting CDP side chains as thick tubes. Non‐bonded interactions are in dash‐lines; H‐bonds are in blue and salt bridges in magenta. c) Ligand interactions diagram of Glc‐1‐P in the donor binding subsite (−1) of CDP. Arrows indicate donor‐to‐acceptor H‐bonds, dash lines H‐bonds with side chains, and solid lines H‐bonds with enzyme backbone. The solid line shaded red to blue represents a salt bridge.

Figure 1a shows the binding epitope mappings of Glc‐1‐P and the other four non‐natural sugar 1‐phosphate ligands analysed. In these maps, different relative normalised STD values on different regions of the ligands report on their distinct spatial contacts (proximities) to the surface of the CDP enzyme in the binding pocket (where higher normalised STD values correspond to closer ligand‐enzyme contacts). For the natural CDP donor substrate, Glc‐1‐P, the epitope mapping shows very close contacts with the enzyme in the bound state all around the glucopyranose ring. All non‐exchangeable protons of Glc‐1‐P showed very high normalised STD values, above 80 % (Figure 1a), supporting a very intimate recognition of Glc‐1‐P by CDP in the donor subsite, which contrasts with the other non‐natural ligands studied, as discussed below. It is worth noticing the large STD value on H1 of Glc‐1‐P, indicating a close recognition by CDP, agreeing well with the known specificity of the enzyme towards the α‐anomer.[^1a^, ^1b^]

Remarkably, the largest saturation transfer is on protons H5 and H6s of the glucopyranose ring, strongly suggesting a major role of the hydroxymethyl group at C5 of Glc‐1‐P for binding to CDP. This STD NMR result matches well with the known limited turnover of Xyl‐1‐P as donor substrate for CDP,[^11^, ^18^] which lacks the hydroxymethyl group at C5. Finally, protons H2 and H4 showed lesser contribution to the recognition of the donor substrate, with H2 showing the lowest STD (Supporting Information Figure S2 and Table S6).

To deepen our structural understanding of the molecular recognition of Glc‐1‐P by CDP, we generated a 3D model of the CDP/Glc‐1‐P complex by protein‐ligand docking (see Materials and Methods and Supporting Information Table S7) and analysed the resulting structure in qualitative terms on the basis of its correlation with the experimental STD NMR data. Figure 1b shows the most populated solution from the docking calculations. To help visualisation of the location and orientation of Glc‐1‐P within the binding site, a superposition of the terminal non‐reducing Glc ring of the published CDP‐bound d‐cellotetraose structure is also shown in Figure 1b.^[11]^ In the docking model, Glc‐1‐P is accommodated in a position compatible with the expected nucleophilic attack, with the phosphate group located in a lobe adjacent to the −1 subsite of the active site as in the crystal structure of cellotetraose‐bound CDP.^[11]^

Figures 1b and c show that the phosphate group of Glc‐1‐P makes H‐bonds with five different amino acids (the side chains of Arg486, Phe815, Gln874, and Ser889, as well as the backbone of Gly890), and a salt bridge with Arg486. The glucopyranose ring is anchored at the binding subsite −1, with hydroxyl groups at C2, C3 and C6 acting as H‐bond acceptors with the side chains of Arg496 and Arg501 and the backbone NH of Asp624, respectively. Additionally, the hydroxyl groups at C3 and C4 act as H‐bond donors with the side chain of Glu502 and the backbone NH of Trp622.

The 3D docking model shows an excellent agreement with the experimental binding epitope mapping from STD NMR experiments (Figure 1a, Supporting Information Table S6). Thus, the glucopyranose ring is intimately contacting CDP, with protons H2 and H4 being more water exposed (Figure 1b) due to a slight tilt of the sugar ring in comparison to the non‐reducing terminal ring of d‐cellotetraose in the published CDP‐bound structure.^[11]^ Remarkably, the hydroxymethyl group is located between Trp622 and Asp624, making close contacts with the Trp622 side chain, explaining the largest transfer of saturation towards protons H6, H6’ and H5 (Supporting Information Table S6). The role of Trp622 side chain as a “hydrophobic platform” has already been described,[^11^, ^19^] and we show here for the first time the atomic details making it a key element for the recognition of the natural donor substrate. In addition, the proximity to Asp624 at the CDP catalytic cleft agrees with its known pivotal role in enzyme catalysis due to its ability to act as proton donor/acceptor in the S_N_2 reaction mechanism.^[19]^

### Impact of epimerisation on CDP binding: Non‐natural donor‐like ligands Gal‐1‐P and Man‐1‐P

The NMR‐validated 3D molecular model of the CDP/Glc‐1‐P complex (Figure 1b) shows that space is available at the binding pocket for some configurational or functional group changes on the hexopyranose ring. This agrees well with the previously reported ability of CDP to use some non‐natural ligands as donor substrates, although with reduced catalytic efficiency (k_cat_/K_M_) in comparison to Glc‐1‐P.^[16a]^ We then decided to explore first the binding of glucose epimers at positions C4 (Gal‐1‐P) and C2 (Man‐1‐P). STD NMR experiments demonstrated that both Gal‐1‐P and Man‐1‐P bind CDP in solution. The binding of Man‐1‐P to CDP in solution is indeed reported here for the first time. In previous works its binding could not be demonstrated yet only suggested by its inhibition of the natural CDP reaction, impacting on the enzymatically produced cellodextrin oligomer length.^[11]^

The binding epitope mapping of Gal‐1‐P (Figure 1a) shows that it makes the closest contact with CDP at the position of H1. The configurational change at C4 relative to Glc‐1‐P gives rise to a rearrangement of the hexopyranose ring, suggested by the reduced saturation transfer to protons H3 and H5 in Gal‐1‐P (Supporting Information Figure S3 and Table S8). In contrast, a configurational change at C2 does not significantly impact the binding mode of the hexopyranose ring by CDP, as deduced from the binding epitope mapping of Man‐1‐P (Figure 1a), which is quite similar to that of Glc‐1‐P, with protons H1, H3 and H6 receiving large saturation transfer, supportive of an intimate recognition by CDP as in the case of Glc‐1‐P (Supporting Information Figure S4 and Table S9).

These results are intriguing as Man‐1‐P is not processed by CDP.[^11^, ^16a^] Our STD NMR study thus shows that a C2 configurational change, although previously demonstrated to be detrimental for catalytic activity, does not impair binding nor affect the binding mode. The null catalytic efficiency for Man‐1‐P cannot be then explained by a change in the binding mode in the donor site, relative to Glc‐1‐P, but rather by the impact of the configurational change at C2 on the network of interactions with the catalytic residues of CDP at the donor binding subsite −1 in the transition state for the CDP‐catalysed reaction.

### Impact of functionalisation on CDP binding: Functionalised donor‐like ligands GlcN‐1‐P and 6F‐Glc‐1‐P

Next, we studied the functionalised glucose analogues GlcN‐1‐P and 6F‐Glc‐1‐P, as it has been previously proved the ability of CDP to use them as active donor substrates.[^11^, ^16b^] The binding epitope mappings of GlcN‐1‐P (Figure 1a and Supporting Information Figure S5 and Table S10) and 6F‐Glc‐1‐P (Figure 1a and Supporting Information Figure S6 and Table S11) revealed close contacts with CDP at the hydroxymethyl groups and less contacts at protons H1 and H2. The introduction of an amine group at C2 (GlcN‐1‐P) results in less contacts of the glucopyranose ring with CDP (Figure 1a), whilst catalytic activity has been reported to be preserved.^[11]^ The reduction at H2 can be explained by a simultaneous steric hindrance and electrostatic repulsion of the amine group with the positively charged side chain of Arg496, located in the −1 subsite of the catalytic cleft (Figure 1b).[^11^, ^16a^] In addition, the close contacts for H6 s in 6F‐Glc‐1‐P indicate an intimate recognition of the fluoromethyl group, supporting tolerance of CDP for a group at position 6 isosteric to OH acting as an H‐bond acceptor. This is in perfect agreement with the recently proved activity of 6F‐Glc‐1‐P as a donor, which has been harnessed for the CDP‐catalysed enzymatic synthesis of multiply 6F‐cellodextrin chains.[^16b^, ^16c^]

Globally, the NMR validated 3D model of the CDP/Glc‐1‐P complex along with the comparison of the STD NMR results for all the non‐natural donor‐like ligands (Figure 1a) provide key structural features to understand the broad molecular recognition ability of CDP towards non‐natural ligands, supporting: (i) the importance of H‐bond acceptor at position 6 of the hexopyranose ring, and (ii) the key relevance of the equatorial hydroxyl at position 4. In the latter case, Gal‐1‐P received the lowest saturation transfer in comparison to the other donor‐like ligands (see Supporting Information Figures S3–S6), what is compatible with a lower binding affinity. Additionally, it showed a significantly different binding epitope. Although STD NMR intensities report on binding rates (k_on_ and k_off_) and not on catalysis (k_cat_), it is worth noting that our binding data correlate well with previous studies where epimerization at C4 led to an increase in the $K_{m}^{app}$ from 3 mM (Glc‐1‐P) to 9.3 mM (Gal‐1‐P), whereas for functionalised Glc‐1‐P analogues, like GlcN‐1‐P, only a slight decrease on the $K_{m}^{app}$ was reported.^[16a]^

### Structural basis of the molecular recognition of natural and non‐natural acceptor‐like ligands

A set of four acceptors/acceptor‐like mono‐, di‐ and trisaccharides: d‐glucose, d‐cellobiose, d‐laminaribiose and d‐cellotriose, were studied to gain structural details about the general molecular recognition of glycans by CDP. STD NMR experiments on d‐glucose did not show any STD signal (Supporting Information Figure S7, a), in good agreement with CDP poor affinity towards this monosaccharide,[^8a^, ^11^] whereas all the other acceptors and acceptor‐like ligands showed STD NMR signals (Supporting Information Figure S7, b, c, d and e).

**Figure 2:**
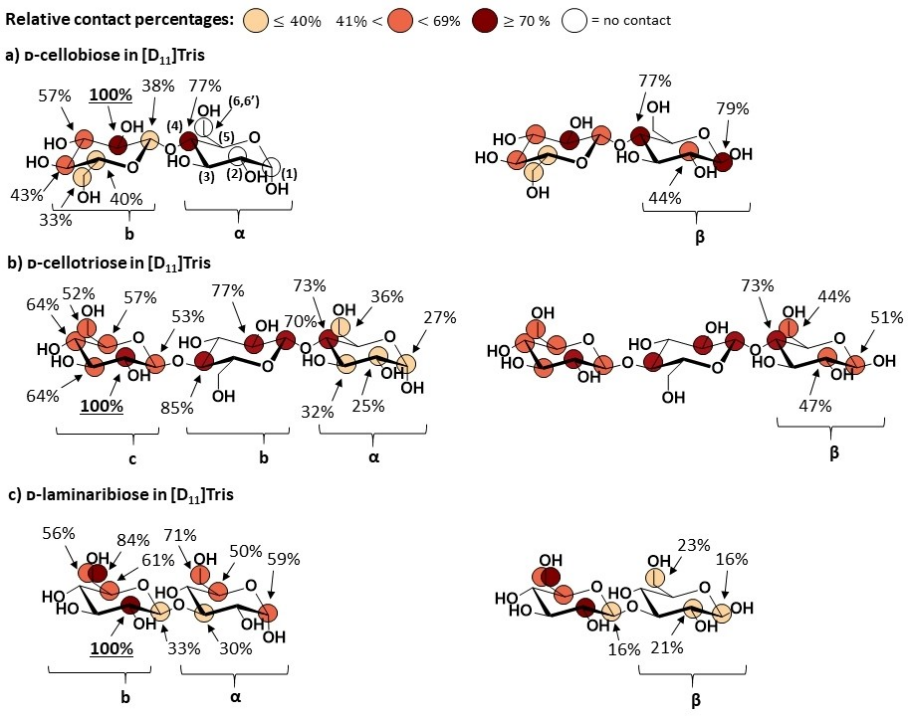
Molecular recognition of natural and non‐natural acceptor‐like ligands by CDP. Binding epitope mappings from STD NMR for the interactions of CDP with a) d‐cellobiose, b) d‐cellotriose and c) d‐laminaribiose. Numbering of the glucopyranose atom positions is reported in d‐cellobiose reducing ring, while the curly brackets below the structures indicate the ring labels. STD NMR experiments were carried out at 800 MHz with samples containing 3 mM ligand and 15 μM enzyme binding sites in [D_11_]Tris 25 mM, pH 7.4 (NaCl 100 mM) at 278 K. Except for all glucose reducing rings and H1 of the non‐reducing ring of d‐laminaribiose, the STD values reported are average values for both α‐ and β‐anomeric forms of the oligosaccharides.

The experimental binding epitope mappings of d‐cellobiose, d‐cellotriose and d‐laminaribiose for their interactions with CDP are shown in Figure 2. Resonances for each proton of the reducing sugar α‐ and β‐anomers were distinguished, and the influence of the anomeric configuration on chemical shifts was further observed up to some resonances at the non‐reducing sugar rings.^[20]^ This allowed us to integrate some isolated resonances for the α‐ and β‐anomeric spin systems of the reducing sugar rings (H1α, H1β, H2α, H2β and H6α for d‐cellobiose and d‐cellotriose; H1α, H1β, H2β, H3α, H5α, H6α, H6’α and H6β for d‐laminaribiose reducing ring) as well as H1b/α and H1b/β for the non‐reducing glucose ring of d‐laminaribiose.

**Natural acceptor substrates: d‐cellobiose and d–cellotriose**. d‐Cellobiose interacts with CDP making close contacts at the non‐reducing glucose ring (Figure 2a, Supporting Information Figure S8 and Table S12). The binding epitope mapping is in excellent agreement with key role of the non‐reducing ring, which is the residue to be cleaved at the enzymatic direct phosphorolysis reaction. The closest contact of d‐cellobiose with the enzyme is made at H2 at that non‐reducing ring (Figure 2a), while reduced contacts are observed progressively from H3 to H6, with the hydroxymethyl group showing the lowest saturation transfer of the whole disaccharide. These results agree with the known inability of CDP to tolerate modifications at the C2 position of the non‐reducing sugar ring of the acceptor as a lack of turnover was shown for mannotriose.^[11]^

Further, in contrast to the glucose ring on Glc‐1‐P, less contacts are observed for d‐cellobiose at the hydroxymethyl group at C5, explaining the reported ability of CDP to recognise xylose‐derivatives as acceptors.^[11]^ Across the β‐(1,4) inter‐glycosidic linkage, significantly larger saturation transfer was observed for H4 at the reducing glucose ring in comparison to the non‐reducing H1. Interestingly, the β‐anomer of the disaccharide received larger amount of saturation at the reducing d‐glucose ring (Figure 2a), with a binding epitope mapping revealing a more intimate contact to CDP in comparison to the α‐anomer; H1β presented the second closest contact with CDP, whereas most of the protons of the α‐anomer did not show contacts at all (Figure 2a).

**Figure 3:**
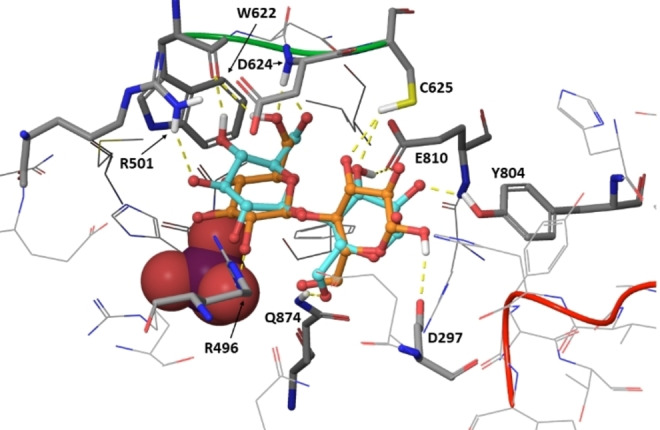
3D complex of CDP with d‐cellobiose. Representation of the best scored docking poses (induced fit docking) for α (light blue) and β (orange) anomers of d‐cellobiose in the acceptor binging pocket of CDP. The amino acid residues establishing interactions with the ligands are represented as thick tubes and labelled, the others are represented as wires. The two d‐cellobiose molecules are represented as ball‐and‐stick. Inorganic phosphate is in pink and represented as CPK.

To rationalise in structural terms the observed differences between α‐ and β‐anomers of d‐cellobiose, we ran molecular docking calculations in the presence of inorganic phosphate (Figure 3 and Supporting Information Table S13). All resulting clusters allocated d‐cellobiose sugar rings in subsites −1 and +1 (Supporting Information Figure S9). The best scored poses were in very good agreement with the STD NMR data, predicting closer contacts at the non‐reducing glucose ring (Figure 3 and Supporting Information Figure S9). Comparing α‐ and β‐anomers, their docking poses resulted in differences in the ψ torsional angle at the inter‐glycosidic linkage (Supporting Information Table S14), explaining well the experimental NMR observations. Only the anomeric hydroxyl group of the β‐anomer of d‐cellobiose establishes a hydrogen‐bond with the side chain of Asp297 (Figure 3), driving the H1β proton closer to the enzyme. This is in excellent agreement with the increased saturation transfer, in contrast to the negligible saturation transfer to H1α proton.

Next, we studied the binding of the longer trisaccharide d‐cellotriose, to extend our investigation to the +2 subsite of the catalytic cleft. Its binding epitope mapping (Figure 2b and Supporting Information Figure S10 and Table S15) showed some similarities with that of d‐cellobiose β‐anomeric spin system, with the closest contact on H2 of the non‐reducing ring and lower saturation transfer to the other protons of the ring. Overall, the central ring showed intimate contacts with the enzyme, with H4 displaying the second closest contact. H1 at the non‐reducing ring showed lower saturation transfer than H4 of the central ring, reporting a recognition of that particular β‐(1,4) inter‐glycosidic linkage similar to d‐cellobiose. However, in contrast to d‐cellobiose, d‐cellotriose showed contacts for both the reducing α‐ and β‐glucose rings, with the β‐anomer showing a more intimate contact with CDP in the bound state.

### Impact of interglycosidic regiochemistry on CDP binding: Non‐natural ligand d–laminaribiose.

To explore the impact of the inter‐glycosidic regiochemistry on CDP binding we compared the binding of d‐cellobiose with its regioisomer d‐laminaribiose (d‐glucose‐β(1,3)‐ d‐glucose). The STD NMR results are shown in Figure 2c (see also Supporting Information Figure S11 and Table S16). Similar to d‐cellobiose, H2 of the non‐reducing glucose ring received the largest saturation transfer. However, in contrast to d‐cellobiose and d‐cellotriose, the closest contact at the reducing ring of d‐laminaribiose was observed for the α‐anomer. Additionally, the good spectral resolution for d‐laminaribiose allowed us to detect the impact of the reducing ring anomeric configuration up to the non‐reducing glucose sugar signals. Thus, H1b of α‐d‐laminaribiose showed significantly higher saturation transfer compared to H1b of β‐d‐laminaribiose. Our results indicate that for the molecular recognition of CDP acceptor regioisomers with a “d *‐glucose‐β‐(1‐X)‐* d *‐glucose*” sequence, CDP shows a preferential recognition for the α‐anomer of those disaccharides with a β‐(1‐3) inter‐glycosidic regiochemistry (d‐laminaribiose), whereas this preference shifts toward the β‐anomer when the inter‐glycosidic regiochemistry is β‐(1‐4) (d‐cellobiose).

The observed differences between anomers prompted us to characterise their bound conformations, to explore if there are concomitant conformational differences upon binding to CDP. We then carried out exchange‐transferred‐NOESY experiments (*tr‐*NOESY) on a sample containing a 1 : 10 CDP: d‐laminaribiose ratio (Supporting Information Figure S12), and bound NOEs were compared to those in the free state. The focus was on the key inter‐glycosidic NOEs, so a quantitative analysis of H1b‐H3α and H1b‐H3β NOEs was performed and key ^1^H‐^1^H distances of the disaccharides in the bound state were derived (Table 1). No significant changes in the ^1^H‐^1^H inter‐glycosidic distances were observed, indicating that the differences in binding epitope mappings for α‐ and β‐anomers are not correlated with a conformational change upon binding to CDP. This result supports that, in contrast to d‐cellobiose, its β‐(1‐3) regioisomer, d‐laminaribiose, must bring the reducing glucose ring closer the surface of the protein in the case of the α‐anomer, without any significant perturbation of the inter‐glycosidic linkage conformation, as a consequence of the distinct orientations of their reducing sugar rings imposed by their differences in inter‐glycosidic linkage regiochemistry.

**Table 1:** Inter‐glycosidic ^1^H‐^1^H distances (Å) of d‐laminaribiose determined from *tr‐*NOESY experiments considering the Isolated Spin Pair Approximation.^[15]^ Cross‐relaxation rates (σ_NOE_) were approximated by the ratio of the normalised NOE volume and the mixing time.

	Free d‐laminaribiose		Bound d‐laminaribiose	
Proton pairs	distance (Å)	*σ*_NOE_	distance (Å)	*σ*_NOE_
H1(b)‐H3(α)	2.94	0.02	2.92	−0.08
H1(b)‐H3(β)	3.20	0.01	3.26	−0.04
H1(b)‐H3(b)	2.66	0.04	2.66	−0.13

### Impact of phosphate on the binding of acceptors

**Figure 4:**
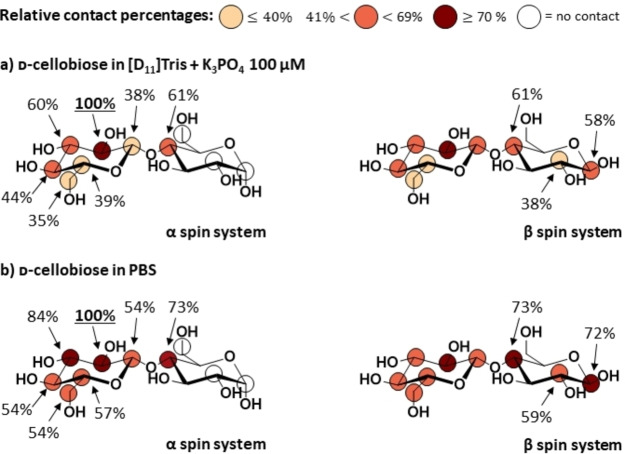
Effect of phosphate on the binding of d‐cellobiose to CDP. Binding epitope mappings derived from the initial slope approach for each isolated proton.^[13]^ a) sample in 25 mM [D_11_]Tris pH 7.4, 100 mM NaCl in the presence of 100 μM K_3_PO_4_, and b) sample in 25 mM PBS pH 7.4, isotonic. The maximum STD_0_ was observed for H2 in the non‐reducing ring, to which an arbitrary value of 100 % was assigned. The STD_0_ of the other protons were normalised against H2.

The phosphate anion is a key player in the phosphorylase reaction. To deepen our detailed understanding of the binding specificity of CDP, we also explored the impact of phosphate on the binding of the acceptor ligands. First, we carried out STD NMR experiments on CDP/ d‐cellobiose samples after addition of 100 μM phosphate (K_3_PO_4,_ Figure 4a, Supporting Information Figure S13 and Table S17). Furthermore, we also carried out STD NMR experiments with a large enough phosphate excess to ensure saturation of the CDP binding pocket, using a 25 mM PBS pH 7.4 buffer (Figure 4b, Supporting Information Figure S14 and Table S18).

Addition of phosphate led to some changes in the binding epitope mapping at the non‐reducing ring in the form of STD increases at protons H3, H4 and H5, particularly at high phosphate excess. This is also in excellent agreement with the 3D models for the binding of d‐cellobiose anomers to CDP (Figure 3), generated in the presence of phosphate. In these models, hydroxyl groups at C2, C3, and C4 sit on top of the anion, bringing H3, H4 and H5 closer to the CDP binding surface, explaining their increase in relative STD values. Globally, however, the binding epitope mapping of d‐cellobiose was preserved upon phosphate titration (cf. Figures 2a and 4), so that the presence of phosphate does not significantly affect the acceptor substrate binding mode further than getting the C2‐C3‐C4 area of the non‐reducing ring a bit closer to the surface of the binding pocket.

Notably, however, addition of phosphate led to a significant decrease in absolute STD NMR intensities of d‐cellobiose (38 % on average) which seemed to equilibrate upon saturation of CDP with phosphate in the 25 mM PBS pH 7.4 sample (30 % on average, Supporting Information Table S19). As no changes were observed in the binding epitope mappings, this reduction in absolute intensities pointed to potential affinity changes for d‐cellobiose in the presence of phosphate.

To investigate this, the apparent dissociation constant (K_D_^app^) for d‐cellobiose binding to CDP was determined from titration experiments with d‐cellobiose in the absence and presence of phosphate (see Materials and Methods). CDP slowly hydrolysed the acceptor in the time scale of tens of hours, precluding a full STD NMR initial slope analysis,^[14]^ so quantification of K_D_ was done by running the STD NMR titration at a single short saturation time (1 s).

**Figure 5:**
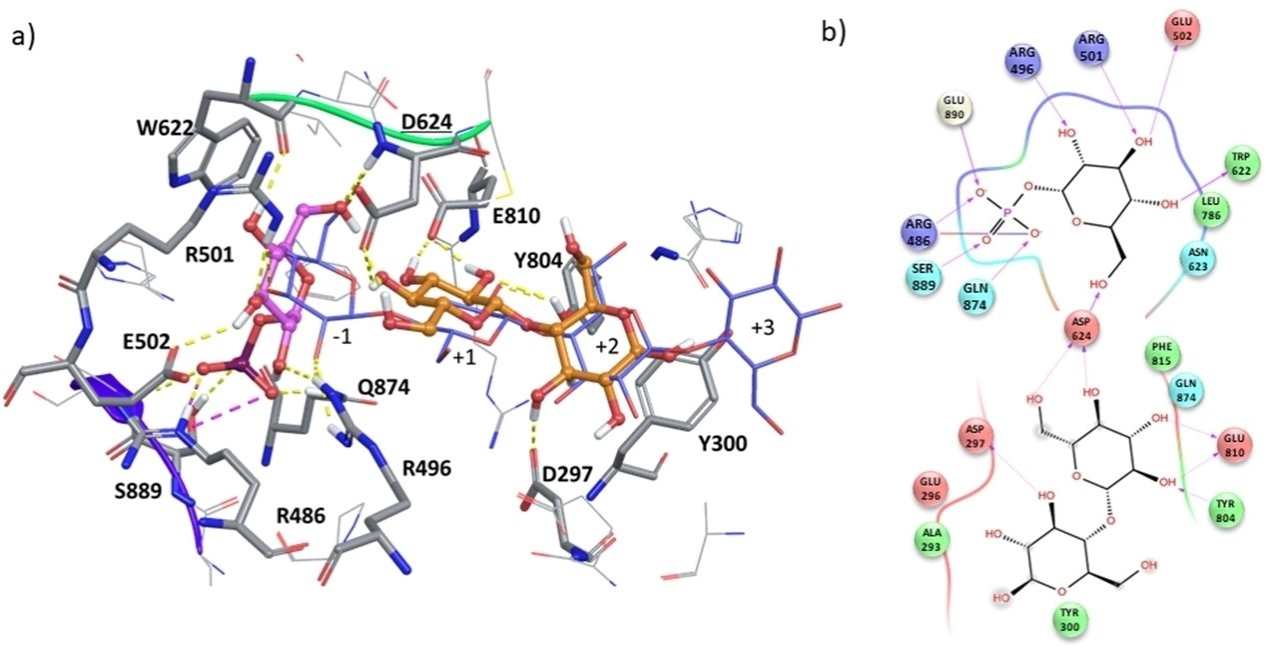
3D ternary complex CDP/Glc‐1‐P/d‐cellobiose from docking calculations. a) The poses of acceptor and donor substrates are compared with the d‐cellotetraose structure from X‐ray crystallography.^[11]^ The main amino acids residues establishing interactions with the substrates are represented as thick tubes; Glc‐1‐P and d‐cellobiose are represented as balls‐and‐sticks in magenta and orange, respectively. b) Ligand interactions diagram of Glc‐1‐P in the donor binding subsite (−1) and d‐cellobiose in the acceptor binding subsite (+1 and +2) of CDP, respectively. Arrows indicate donor‐to‐acceptor H‐bonds, dash lines H‐bonds with side chains, and solid lines H‐bonds with enzyme backbone. The solid line shaded red to blue represents a salt bridge.

K_D_^app^ values were determined from the proton of d‐cellobiose with the lowest STD absolute intensity, to avoid impact of differential relaxation times on the K_D_^app^ determination.^[14]^ Thus, we monitored the titration via the amplification factor (STD‐AF) of H4_β_ under three different experimental conditions: (i) absence of phosphate ([D_11_]Tris 25 mM, pH 7.4, NaCl 100 mM; (ii) 10‐fold excess of phosphate to binding sites, and (iii) very large excess of phosphate (PBS 25 mM pH 7.4, isotonic). Table 2 shows the different K_D_^app^ values, proving that the cofactor plays a role in the affinity of the substrate producing a slight increase in acceptor binding affinity, explaining the observed differences in STD NMR intensities (Figure 5). These experiments highlight the exquisite sensitivity of STD NMR intensities to changes in affinities under the conditions tested.

**Table 2:** Apparent equilibrium dissociation constant K_D_^app^ [mM] calculated from STD NMR titration. The STD‐AF (amplification factor) from proton H4β was followed for d‐cellobiose bound to CDP in [D_11_]Tris buffer 25 mM pH 7.4 NaCl 100 mM, [D_11_]Tris buffer 25 mM pH 7.4 NaCl 100 mM in presence of K_3_PO_4_ and PBS buffer 25 mM pH 7.4, isotonic.

	[D_11_]Tris	[D_11_]Tris with K_3_PO_4_	PBS
K_D_ (H4β), mM	2.2 (±0.7)	2.0 (±1.1)	1.2 (±0.7)

As phosphate does not impact the acceptor binding mode yet it slightly affects the affinity, we additionally investigated if the presence of phosphate influences the bioactive conformation of the disaccharide. Tr‐NOESY experiments were performed to characterise the conformation of d‐cellobiose bound to CDP. Cross relaxation rates (σ_NOE_) were approximated by the ratio of the normalised NOE volume and the mixing time (see Supporting Information), and the Isolated Spin Pair Approximation was used to calculate distances.^[15]^ The results (Supporting Information Table S20) demonstrate that the conformation around the β‐(1‐4) linkage does not significantly change neither upon binding to CDP, nor after addition of phosphate in the bound state (2D‐NOESY spectra in Supporting Information Figure S15).

Globally, our study shows that binding of phosphate favours the binding of the acceptor without changing its binding mode. This result supports our 3D docking model (Figure 3) where the non‐reducing d‐cellobiose sugar ring in −1 subsite closes off the binding site lobe where phosphate is located. This is similar to the published d‐cellotetraose bound CDP X‐ray structure, that suggested a sequential Bi Bi mechanism where phosphate must be bound before the glucan co‐substrate in the phosphorolytic reaction.^[11]^

### Structural details of the interactions in the CDP/Glc‐1‐P/d‐cellobiose ternary complex

To finally get a 3D structural understanding of the molecular recognition of substrates by CDP, we generated a 3D model of the ternary complex CDP/Glc‐1‐P/ d‐cellobiose by protein‐ligand docking. The calculations were performed by docking d‐cellobiose onto our previously obtained structure of CDP bound to Glc‐1‐P (Supporting Information Table S21), and the most populated solution is shown in Figure 5.

The resulting structure was analysed on the basis of its correlation with the published CDP‐bound d‐cellotetraose structure.^[11]^ To help visualisation of the location and orientation of the donor Glc‐1‐P and acceptor d‐cellobiose substrates within the binding site, a superimposition of the published CDP‐bound d‐cellotetraose structure is also shown in Figure 5.^[11]^

The 3D docking model of the ternary complex shows that d‐cellobiose enters the +1 subsite by the non‐reducing ring, an orientation compatible with the reverse phosphorolysis mechanism. The residue Asp624 has a bridge function between donor and acceptor substrates, with the hydroxymethyl group of Glc‐1‐P acting as H‐bond acceptor of the backbone NH of Asp624, and the hydroxyl groups at C4 and C6 of d‐cellobiose acting as H‐bond donors to the Asp624 side chain (Figure 5).

The hydroxyl groups at C2 and C3 of the non‐reducing ring of d‐cellobiose act as H‐bond donors to the side chain of Glu810, while the hydroxyl group at C2 acts as H‐bond acceptor of the OH of the side chain of Tyr804. Additionally, the reducing ring of d‐cellobiose establishes a favourable CH‐π stacking interaction with Tyr300 in the +2 subsite.

This CH‐π stacking interaction explains well the STD binding epitope mapping of d‐cellotriose (Figure 2b), where a more intimate contact was reported for the reducing ring occupying the +2 subsite of the β‐anomer in comparison to the α‐anomer. A configurational change of the anomeric proton from β‐ to α‐ can lead to a disruption of the CH‐π stacking interaction, causing a reduction in the enthalpic contribution for the binding event.

Comparing the 3D docking model of ternary complex CDP/Glc‐1‐P/d‐cellobiose with the available literature on cellobiose phosphorylase from other organisms like *Cellovibrio gilvus* (CBP) and *Clostridium stercorarium*, important structural information can be gained. In the first case, CBP crystal structure (PDB: 3QG0)^[21]^ showed that the enzyme misses a residue key to establish a CH‐π stacking interaction in the +2 subsite. This observation can explain the ability of CBP to synthetize disaccharides but no longer oligosaccharides chains, supporting the hypothesis that this residue is pivotal for the productive binding of d‐cellobiose and longer saccharides. In the second case, previous studies on CDP from *Clostridium stercorarium* ^*[22]*^ reported the presence of a Trp residue in the +2 binding subsite. Mutation to Ala residue in place of the Trp resulted in retention of 50 % activity on recognising d‐cellobiose as acceptor, indicating the contribution of other residues at the +2 subsite in the substrate recognition.

Overall, the results from this study have allowed us to gain information on the structural basis behind the broad molecular recognition ability of cellodextrin phosphorylase from *Ruminiclostridium thermocellum* (CDP) towards different natural and non‐natural donor‐ and acceptor‐like ligands. The impact of ligand stereochemistry, regiochemistry, functionalisation, as well as the impact of phosphate on the molecular recognition have been studied by NMR, and the structural data demonstrate the broad breadth of ligand molecular patterns that can be recognised by CDP.

## Conclusion

A thorough study by a combination of NMR spectroscopy and molecular docking calculations has allowed us to understand the structural basis for the broad molecular recognition ability of cellodextrin phosphorylase (CDP) from *Ruminiclostridium thermocellum* towards donor‐ and acceptor‐like ligands in solution. We have expanded the knowledge of the molecular details of the binding of natural (Glc‐1‐P, d‐cellobiose and d‐cellotriose) and non‐natural ligands (Gal‐1‐P and Man‐1‐P, glucose analogues GlcN‐1‐P and 6F‐Glc‐1‐P, and the cellobiose regioisomer d‐laminaribiose) to the ‐1, +1 and +2 subsites of the enzyme. CDP recognises all the investigated donor and donor‐like ligands, even those for which previous studies have reported impairment of the enzyme catalytic efficiency.[^11^, ^16a^] Structural analysis of Glc‐1‐P binding revealed a close recognition of the hexopyranose ring in the ‐1 subsite of CDP. On the other hand, Gal‐1‐P, Man‐1‐P and 6F‐Glc‐1‐P show a different binding epitope at the hexopyranose ring upon binding to CDP, in comparison to Glc‐1‐P. Man‐1‐P binds CDP with a global binding mode similar to Glc‐1‐P, proving that configurational changes at C2 level do not impair binding. Our results on acceptor/acceptor‐like ligands indicate CDP selectivity towards α‐ and β‐anomeric configuration in the cases of β‐(1‐3) and β‐(1‐4)‐oligosaccharide ligands, respectively. In addition, our data indicate that the area around C2 of the non‐reducing glucose ring is a crucial contact for the recognition of acceptors. We have also revealed the role played by inorganic phosphate on acceptor substrate recognition by enhancing binding affinity. Finally, we provide an NMR validated molecular docking 3D model of the CDP/donor/acceptor ternary complex, which allows to understand structural details of the binding of both substrates in the reverse phosphorolysis reaction.

In summary, this work provides valuable structural information on the molecular recognition in solution of natural and non‐natural ligands by a cellulose producing enzyme, CDP, confirming, in structural terms, its ability to accommodate chemically diverse donor‐ and acceptor‐like substrates in the binding pocket, which makes this enzyme appropriate for the preparation of chemically modified cellulose‐like polysaccharides, of strong potential for the design and engineering of novel functionalised cellulose‐based biomaterials of biotechnological interest.

## Experimental Section

**Protein expression and exchange**: CDP enzyme (1009 amino acids, 114 kDa per monomer; CDP is a homodimer), was expressed as previously reported.[^11^, ^16b^] Deuterated solvent exchange ([D_11_]Tris 25 mM, pH 7.4, 100 mM NaCl) was performed via 50 kDa MWCO filter, centrifuge 4000 rfm, 4 °C, 5 cycles of 20 minutes each. The final concentration of the protein was measured with a Thermo Scientific NanoDrop UV‐Vis spectrophotometer using A_280_ (ϵ=117.635 (ϵx1000 set up)). The concentrated protein was diluted to the desired concentration for NMR analysis using [D_11_]Tris 25 mM, pH 7.4, 100 mM NaCl buffer.

**Nuclear magnetic resonance**: ^1^H and ^13^C resonance assignment for ligands was performed via 1D ^1^H NMR, 2D ^1^H,^1^H DQF‐COSY, ^1^H,^13^C HSQC and ^1^H,^1^H NOESY experiments in [D_11_]Tris 25 mM, pH 7.4 100 mM NaCl at 278 K.

### Saturation transfer difference (STD) NMR and binding epitope mapping

**Donor‐like ligands**: All STD NMR experiments were carried out on an Avance Bruker 800.23 MHz spectrometer equipped with a 5 mm inverse triple‐resonance probe. The Glc‐1‐P sample consisted of 3 mM ligand and 15 μM in binding sites in [D_11_]Tris buffer (25 mM, pH 7.4, NaCl 100 mM), for a ligand‐to‐enzyme ratio of 200 : 1. For the non‐natural donors (Gal‐1‐P, Man‐1‐P, GlcN‐1‐P and 6‐F‐Glc‐1‐P) the ligand‐to‐enzyme ratio was reduced, hence increasing the fraction of bound ligand (f_LB_) and in turn the STD intensity, using 5 mM of ligand and 50 μM in binding sites instead (100 : 1 ligand‐to‐binding site ratio). **Acceptors** : Samples were prepared using a 200‐fold excess of ligand over binding sites (3 mM ligand, 15 μM enzyme monomers) in [D_11_]Tris buffer (25 mM, pH 7.4, NaCl 100 mM). Phosphate titration experiments with d‐cellobiose were performed by adding, on top of a sample with the above conditions, 100 μM K_3_PO_4_ solution in [D_11_]Tris buffer. In addition, STD NMR experiments in PBS (25 mM, pH 7.4, 150 mM NaCl) were run for d‐cellobiose under the same experimental conditions. STD NMR build‐up curves were acquired at different saturation times (0.5, 0.75, 1, 1.5, 2, 3, 4, 5 and 6 s). Irradiation frequencies were 0.3 ppm and 50 ppm for the *on‐resonance* and the *off‐resonance* spectra respectively. Cascades of 50 ms Gaussian‐shaped pulses at a field strength of 50 Hz were employed, with a delay of 1 ms between successive pulses. The broad protein signals were removed using a 40 ms spinlock (T1ρ) filter (as implemented in the Bruker sequence *stddiff.3*).

Build‐up curves were fitted to a mono‐exponential function (Eq. 1)

(1) $$\;STD\left(t_{sat}\right)=STD^{max}\left(1-e^{-k_{sat}\;t_{sat}}\right)$$

where STD^max^ represents the maximum of the curve, k_sat_ is a rate constant (in s^‐1^) and t_sat_ is the saturation time in seconds. From these STD build‐up curves, we mapped out the main contacts of the ligands to CDP in the bound state by determining the initial slopes (STD_0_) of the curves, obtained as the product of the STD^max^ and k_sat_ coefficients, and thereafter normalising all the STD_0_ values within a given ligand by the highest one, to which an arbitrary value of 100 % was assigned. Different mapping ranges were used for the binding epitopes of donor and acceptor molecules: the donor is buried inside the binding pocket and therefore receives higher saturation transfer and all protons receive significant amount of saturation, whereas in the case of the more loosely recognised acceptor ligands, the gap in STD intensity between the largest and the smallest value is much higher. For accuracy, only well‐resolved NMR resonances for each investigated ligand (e. g., H1β of the reducing ring) were considered in the analysis. The contacts of non‐isolated protons (overlapping signals) are then not reported.

**Transferred‐NOESY (tr‐NOESY)**: Tr‐NOESY experiments were carried out using a phase sensitive pulse programme with gradient pulses in the mixing time and a relaxation delay of 1.5 s. d‐cellobiose was analysed using [D_11_]Tris 25 mM buffer pH 7.4 NaCl 100 mM and a protein/ligand ratio of 1 : 20 at 298 K. Experiments at different mixing times (40 and 160 ms) were collected for the free and bound states. Finally, the same experiments were collected in the presence of a 5‐fold excess of inorganic phosphate. For the binding of d‐laminaribiose at 290 K, a protein/ligand ratio of 1 : 10 was employed. Experiments at different mixing times (40 and 300 ms) were collected in the free and bound state. Experimental ligand distances in the bound state were derived using the isolated spin pair approximation (ISPA) approach.^[15]^ First, each cross peak was divided by its corresponding diagonal peak at 40 ms, thus obtaining the normalized NOE volume. Each volume was then divided by the mixing time to get a good approximation of the cross‐relaxation rate (σ_NOE_). Finally, using the fixed H1‐H5 and H1−H3 distances of the non‐reducing ring terminal (2.38 Å and2.66 Å) for d‐cellobiose and d‐laminaribiose, respectively, the key inter‐glycosidic proton−proton distances were calculated according to Equation 2

(2) $$d_{x}=d_{ref}{\left(\frac{\sigma_{ref}^{NOE}}{\sigma_{x}^{NOE}}\right)}^{1/6}$$

where d_x_ is the unknown distance to be determined, d_ref_ is the distance used as reference, and $\sigma_{ref}^{NOE}$ and $\sigma_{x}^{NOE}$ are the cross‐relaxation rates of the reference and unknown distances, respectively.

**Determination of the apparent dissociation constant** $K_{D}^{app}$ : Ligand binding affinity was measured by STD NMR experiments.[^14^, ^17^] ^1^H STD NMR spectra of d‐cellobiose were acquired at different ligand concentration (0.25, 0.5, 1, 2.5, and 5 mM) with a saturation time of 1 second. *On‐resonance* and *off‐resonance* frequencies were 0.3 and 50 ppm, respectively. A total of 128 number of scans were collected for each experiment, and a relaxation delay of 6 s was employed. Tree different experimental conditions were investigated: 1) absence of phosphate, with [D_11_]Tris 25 mM, pH 7.4 NaCl 100 mM, 2) concentration of phosphate 10‐fold per binding site, 3) large excess of phosphate, with PBS 25 mM pH 7.4, isotonic. The STD intensities were corrected by the excess of ligand to indirectly obtain information about the protein‐ligand complexes concentration in solution (STD amplification factor, STD‐AF). To obtain the K_D_ values, the obtained Langmuir isotherms were fitted to Equation 3, ^[14]^

(3) $$STD-AF\left(\left[L\right]\right)=\frac{\alpha_{STD}\left[L\right]}{K_{D}+\left[L\right]}$$

**Molecular docking**: All calculations were performed using the Schrödinger molecular modelling suite. The crystal structure of the complex of CDP with d‐cellotetraose (PDB code 5NZ8)^[11]^ was processed using the protein preparation wizard.^[23]^ Conformational sampling of α‐d‐glucose‐1‐phosphate (Glc‐1‐P) and d‐cellobiose was performed by a conformational search (MacroModel) based on Monte Carlo Multiple Minimum method in order to enhance the sampling of conformations. In the case of Glc‐1‐P, the obtained poses (13 in total) were used to run docking SP (Glide) with a receptor grid of 10 Å inner box and 20 Å outer box, a 4‐fold enhanced conformational sampling and OPLS3 as force field. In addition, sampling of ring conformations was switched off and the calculation was run in the absence of phosphate in the binding pocket. The obtained conformers were clustered by atomic RMSD and the energetically most favourable pose within the most populated cluster (which showed a good overlapping with the nonreducing ring of d‐cellotetraose complexed with CDP) was selected for analysis and further docking calculations. For d‐cellobiose, a separate conformational search was performed for both the α‐ and β‐anomers. In both cases, the obtained poses were clustered (RMSD) and a representative of the most populated cluster was selected for further calculations. Flexible induced fit docking (IFD) was performed with a 0.8 Å tolerance constrain on the inter‐glycosidic linkage referenced to d‐cellotetraose complexed with CDP. This constraint was introduced as previous attempts led to distortion of the inter‐glycosidic linkage conformation, with ϕ and ψ angles values not allowed in the β‐(1‐4)‐ Carbohydrate Ramachandran Plot reported by GlycoMapsDB (*Glycosciences.de*).^[24]^ The obtained poses were clustered (RMSD). For the α‐anomeric configuration, the most energetically favourable pose of the most populated cluster was saved, whereas in the case of the β‐anomeric configuration we had to discard the first two most populated clusters as the first presented an inverse orientation of the ligand in the binding pocket and the second presented forbidden values of ϕ and ψ angles. Finally, the selected Glc‐1‐P (obtained from the first docking stage) was introduced in the CDP structure to dock a second d‐cellobiose molecule, allowing us to obtain the docking model for the CDP/Glc‐1‐P/d‐cellobiose ternary complex. The selection of d‐cellobiose as second docked substrate was led by its ability act as acceptor in the reverse phosphorolysis reaction (indeed, d‐cellobiose is the natural disaccharide acceptor). In addition, the closer contact for the β‐anomer shown by STD NMR experiments, as well as the additional H‐bonding reported from docking calculations, pointed us to select this configuration for our studies. Docking SP was run for all the obtained poses of d‐cellobiose conformational search, a 4‐fold enhanced conformational sampling and a grid box of 10 Å inner box and 23 Å outer box.

## Conflict of interest

The authors declare no conflict of interest.

## Supporting information

As a service to our authors and readers, this journal provides supporting information supplied by the authors. Such materials are peer reviewed and may be re‐organized for online delivery, but are not copy‐edited or typeset. Technical support issues arising from supporting information (other than missing files) should be addressed to the authors.

Supporting Information
