# Supplementary material for: Molecular Recognition of Natural and Non‐Natural Substrates by Cellodextrin Phosphorylase from Ruminiclostridium Thermocellum Investigated by NMR Spectroscopy
Source: Chemistry. 2021 Oct 8;27(63):15688–98. doi: 10.1002/chem.202102039 (PMC9293210; doi:10.1002/chem.202102039)
Supplement: Supplementary file 1 — Supporting Information [file CHEM-27-15688-s001.pdf]

Chemistry–A European Journal

Supporting Information

Molecular Recognition of Natural and Non-Natural Substrates by Cellodextrin Phosphorylase from *Ruminiclostridium Thermocellum* Investigated by NMR Spectroscopy

Valeria Gabrielli, Juan C. Muñoz-García, Giulia Pergolizzi, Peterson de Andrade, Yaroslav Z. Khimyak, Robert A. Field, and Jesús Angulo*

Author Contributions

V.G. Data curation:Lead; Formal analysis:Lead; Investigation:Lead; Writing – original draft:Lead; Writing – review & editing:Equal
G.P. Data curation:Supporting; Formal analysis:Supporting; Investigation:Supporting; Writing – review & editing:Equal
P.d. Formal analysis:Supporting; Writing – review & editing:Equal
Y.K. Formal analysis:Supporting; Resources:Supporting; Supervision:Equal; Writing – review & editing:Equal
R.F. Formal analysis:Supporting; Resources:Supporting; Supervision:Equal; Writing – review & editing:Equal
J.A. Conceptualization:Lead; Formal analysis:Supporting; Funding acquisition:Lead; Investigation:Supporting; Methodology:Supporting; Supervision:Lead; Writing – original draft:Equal; Writing – review & editing:Lead

Supporting Information Table of content

1.1. **CDP specificity and catalytic kinetic efficiency towards donor and acceptor substrates**

1.2. **Structural basis of molecular recognition of natural and unnatural donor substrates**

1.2.1. **CDP-Donors binding**

1.2.2. **Glucose-1-phosphate**

1.2.3. **Galactose-1-phosphate**

1.2.4. **Mannose-1-phosphate**

1.2.5. **Glucosamine-1-phosphate**

1.2.6. **6-Deoxy-6-Fluorine-Glucose-1-phosphate**

1.3. **Structural basis of molecular recognition of natural and unnatural acceptor-like substrates**

1.3.1. **CDP-Acceptors binding**

1.3.2. **D-cellobiose**

1.3.3. **D-cellotriose**

1.3.4. **D-laminaribiose**

1.4. **Impact of phosphate on acceptor binding**

1.4.1. **Determination of acceptor binding epitope upon phosphate titration**

1.4.2. **Determination of D-cellobiose bioactive conformation upon phosphate titration**

1.4.3. **Structural details of the interactions in the CDP/Glc-1-P/D-cellobiose ternary complex**

1.1. CDP specificity and catalytic kinetic efficiency towards donor and acceptor substrates

Table S1: CDP specificities towards donor-like substrates.

**Donors**
α-D-glucosyl 1-fluoride[1]
α-D-xylose 1-phosphate[2, 3]
α-D-galactose 1-phosphate[3, 4]
α-D-glucosamine 1-phosphate[3, 4]
α-D-galactosamine 1-phosphate[4]
α-D-6-deoxy-6-fluoro-glucose 1-phosphate[5]

Table S2: CDP specificities towards acceptor and acceptor-like substates. The red frame indicates D-glucose or β-D-glucoside derivates as carbohydrate moiety of the acceptor-like substrates (continuous next page).

**Acceptors**
D-Glucose[6-14]
Radioactive D-Glucose[15]
1-Thio-β-D-glucose[16]
1-Azido-1-deoxy-β-D-glucopyranoside (β-glucosyl azide)[8]
2-Azidoethyl β-D-glucopyranoside[17]
Oligo(ethylene glycol) (OEG) bearing β-D-glucose[18]
Alkyl β-D-glucoside[19]
2-aminoethyl-β- D -glucoside[20]
2-(glucosyloxy)ethyl methacrylate[21]
Vinyl glucosides[22]
4-*O*-β-D-Glucopyranosyl-D-glucose (cellobiose)[1, 6, 7, 12, 23-25]
Methyl β-cellobioside[6]

Table S2: continued

Phenyl β-cellobioside[6]
Benzophenonel β-cellobioside[6]
4-Thiocellobiose[6]
Methyl 4-thio-α-cellobioside[6]
Methyl*O*-β-D-glucopyranosyl-(1→3)-S-β-D-glucopyranosyil-(1→4)-*O*-(4-thio-β-D-glucopyranosyl)-(1→4)-β-D-glucopyranoside[26]
N,N-bis(β-D-cellobiosyl)succinamide[27]
Tris(aminoethyl N-carbonylmethyl β-D-cellobiosyl)amine[27]
β-D-cellobiosyl polyamidoamide (PAMAM) dendrimers[27]
2-*O*-β-D- Glucopyranosyl-D-glucose (sophorose)[1]
4-*O*-β-D- Glucopyranosyl-D-altrose[28]
4-*O*-β-D-Glucopyranosyl-D-2-deoxy- D-glucose[28]
4-*O*-β-D- Glucopyranosyl-D-mannose[28]
4-*O*-β-D- Glucopyranosyl-D-xylose[2, 28]
4-*O*-β-D- Glucopyranosyl-1-deoxynojirimytin[29]
4-*O*-β-D-Glucopyranosyl-D-2-deoxy- D-glucose[28]
4-*O*-β-D- Glucopyranosyl-D-mannose[28]
4-*O*-β-D- Glucopyranosyl-D-xylose[2, 28]
4-*O*-β-D- Glucopyranosyl-1-deoxynojirimytin[29]
β-D-2-amine-glucopyranosyl-β-(1→4)-D-glucopyranosyl-β-(1→4)-D-glucopyranose[30]
4-*O*-β-D-Xylopyranosyl-D-xylose (xylobiose)[2]
4-*O*-β-D- Xylopyranosyl-D-glucose[2]
Cellobitol[28]

Table S3: List of tested monosaccharides, disaccharides and trisaccharides acceptors for which CDP Glc-1-P did not show any turn-over. In addition, the only donor-like substrate which did not show any catalytic efficiency is reported in red.

Xylose[1, 31]	N-Acetyl-glucosamine*[1]	Mannobiose*[1]
L-Rhamnose*[1]	β-methyl glucoside[31]	Sucrose*[1]
Salicin[31]	Melibiose*[1]	Talose*[1]
Lactulose*[1]	Isomaltose*[1]	L-Fucose*[1]
Maltose[1, 31]	Mannotriose[3]	N-Acetyl-mannosamine*[1]
Arabinobiose*[1]	Galactose*[1]	Lactose[1, 31]
Xylose[1, 31]	Fructose*[1]	Trehalose*[1]
Mannose*[1]	N-Acetyl-galactosamine*[1]	Turanose*[1]
Arabinose*[1]	Gentibiose[1, 31]	Mannose-1-phosphate[3, 4]

* The acceptor specificity was determined exclusively at 4.4 mM CDP concentrations for 3 h using 200 mM a-Glc1-P and 200 mM of the tested acceptor at pH 5.4 and 40 °C.

Table S4: Kinetics parameters of CDP donor substrates reported towards the synthetic direction. Donor specificity was determined by the measurement of phosphate release keeping the acceptors concentration constant (5 mM). Table reproduced from Singh *et al.*[4]

		**Reverse phosphorolysis**		
**Donors**	**Acceptor**	k_{cat} **(s^{-1})**	K_M^{app} **(mM)**	k_{cat}/K_M^{app} **(mM^{-1}s^{-1})**
**Glc-1-P**	D-cellobiose	16.4 ± 0.7	3.0 ± 0.6	5.5
	D-laminaribiose	15.9 ± 0.4	3.0 ± 0.3	5.3
**Gal-1-P**	D-cellobiose	0.6± 0.02	9.3 ± 1.1	0.06
	D-laminaribiose	0.6± 0.01	10.7 ± 0.7	0.06
**GlcN-1-P**	D-cellobiose	0.08± 0.003	1.6± 0.2	0.05
	D-laminaribiose	0.14± 0.013	5.1± 1.2	0.03
**Man-1-P**	D-cellobiose	NA	NA	NA
	D-laminaribiose	NA	NA	NA

Table S5: Kinetics parameters of CDP acceptor substrates reported towards the synthetic direction. Acceptor specificity was determined by the measurement of phosphate release keeping Glc-1-P concentration constant (10 mM).

Acceptors	Reverse phosphorolysis k_{cat} (s^{-1})	K_M^{app} (mM)	k_{cat}/K_M^{app} ($mM^{-1}s^{-1}$)
**Glucose**[3, 7, 23]	NA/nd	NA/nd	NA/nd
**Phenylβ-D-glucopyranoside**[3]	15.0 ±6.3	24 ±13	0.63
**Cellobiose**[3]	17 ±0.50	2.6 ±0.18	6.5
**Cellotriose**[3]	9.5 ±0.35	0.68 ± 0.076	14
**Cellotetraose**[3]	5.0 ±0.25	0.54 ± 0.13	9.3
**Cellopentaose**[3]	4.3 ±0.47	0.36 ± 0.076	12
**Cellohexaose**[3]	7.6 ±1.8	1.9 ±0.76	4.0

NA not applicable; nd not determined

1.2. Structural basis of molecular recognition of natural and unnatural donor substrates

1.2.1. STD NMR spectra of CDP-Donors binding

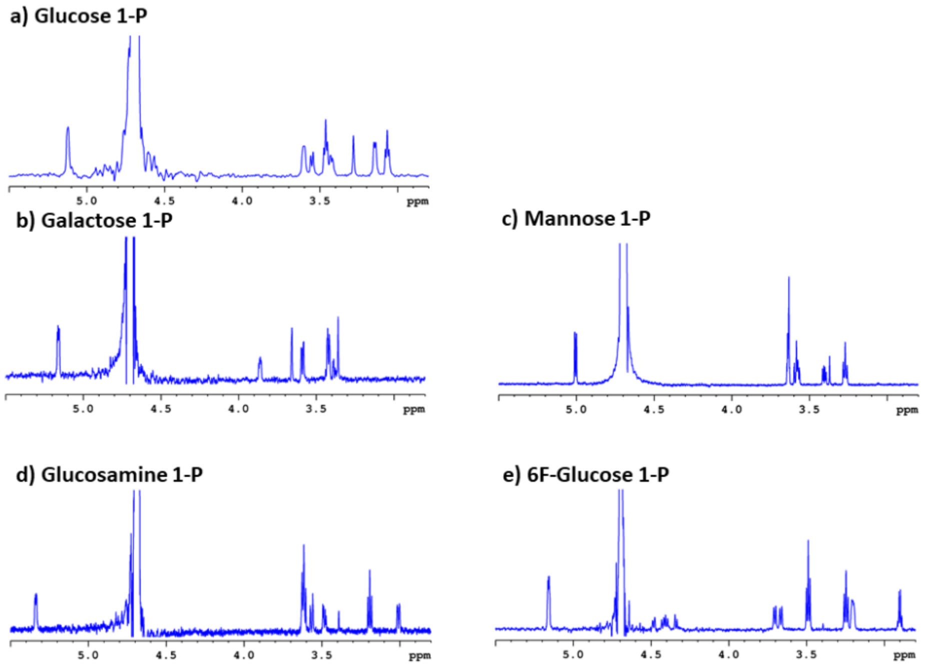

Figure S1: STD NMR difference (off-resonance – on-resonance) spectra for a) Glucose-1-P (Glc-1-P), b) Galactose-1-P (Gal-1-P), c) Mannose-1-P (Man-1-P), d) Glucosamine-1-P (Gln-1-P) and e) 6F-Glucose-1-P recorded at 6 s saturation time in [D_{11}]Tris buffer 25 mM, pH 7.4 (NaCl 100 mM) at 278 K. STD NMR experiments were carried out with samples containing 1:200 "binding site to ligand ratio" for Glc-1-P, and 1:100 ratio for Gal-1-P, Man-1-P, GlcN-1-P and 6F-Glc-1-P. The presence of signals in the difference spectra reveals binding of the studied donors to CDP.

1.2.2. Glucose-1-phosphate (Glc-1-P)

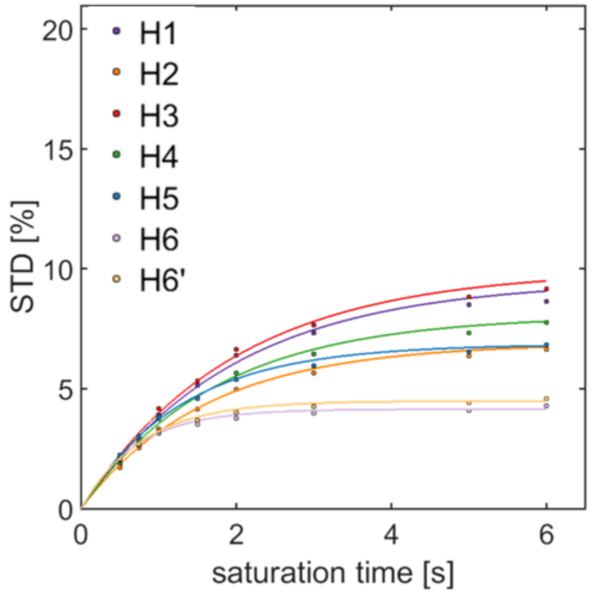

Figure S2: STD build-up curves (symbols: experimental data; solid lines: mathematical fitting to a monoexponential equation) for Glc-1-P in [D_{11}]Tris buffer 25 mM, pH 7.4 (NaCl 100 mM) at 278 K. 15 µM binding unit was used for a ligand concentration of 3 mM.

Table S6: STD^{max}, k_{sat} and STD_0 for Glc-1-P. Relative STD (%) were obtained through normalisation against the largest ligand STD initial slope (H6; 100%)

	STD^{max}	k_{sat}	STD_0	STD (%)
**H1**	9.70	0.52	5.06	91
**H2**	7.01	0.64	4.45	80
**H3**	9.95	0.54	5.34	96
**H4**	8.12	0.59	4.80	86
**H5**	7.03	0.77	5.42	98
**H6**	4.31	1.29	5.56	100
**H6'**	4.77	1.15	5.48	98.5

Table S7: CDP/Glc-1-P complex 3D docking clusters rank and population. The total number of poses obtained by SP docking was 140. The computed binding energy (Glide emodel) and the RMSD refer to the most energetically favourable pose obtained for each cluster.

Cluster rank	Cluster population (%)	Glide emodel (kcal/mol)	RMSD (Å)
1	37	-79.44	0.40
2	21	-79.82	0.43
3	14	-79.36	0.41
4	14	-80.11	0.41
5	14	-77.39	0.42

1.2.3. Galactose-1-phosphate (Gal-1-P)

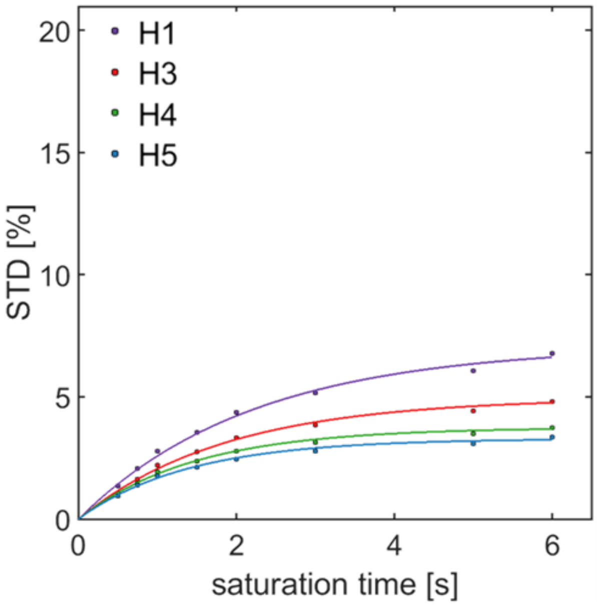

Figure S3: a) STD build-up curves (symbols: experimental data; solid lines: mathematical fitting to a monoexponential equation) for Gal-1-P in [D_{11}]Tris buffer, pH 7.4 (NaCl 100 mM) at 278 K. 50 µM binding unit was used for a ligand concentration of 5 mM.

Table S8: STD^{max}, k_{sat} and STD_0 for Gal-1-P. Relative STD (%) were obtained through normalisation against the largest ligand STD initial slope (H1; 100%)

	STD^{max}	k_{sat}	STD_0	STD (%)
**H1**	7.09	0.45	3.21	100
**H3**	4.96	0.54	2.65	83
**H4**	3.75	0.68	2.54	79
**H5**	3.29	0.71	2.35	73

1.2.4. Mannose-1-phosphate (Man-1-P)

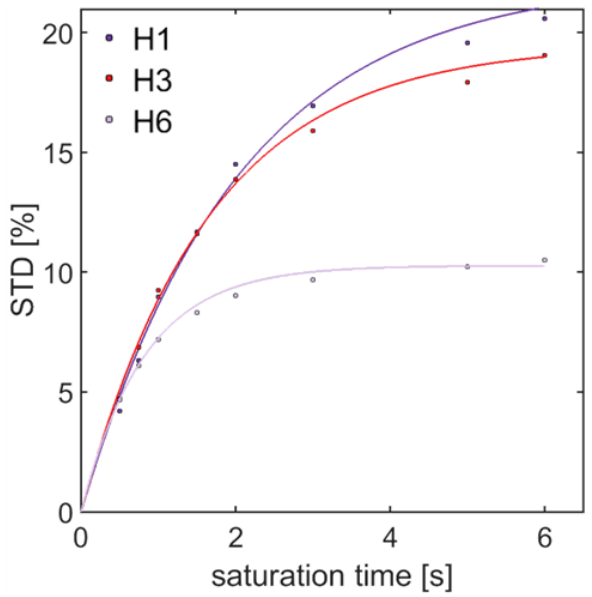

Figure S4: a) STD build-up curves (symbols: experimental data; solid lines: mathematical fitting to a monoexponential equation) and for Man-1-P in [D_{11}]Tris buffer, pH 7.4 (NaCl 100 mM) at 278 K. 50 µM binding unit was used for a ligand concentration of 5 mM.

Table S9: STD^{max}, k_{sat} and STD_0 for Man-1-P. Relative STD (%) were obtained through normalisation against the largest ligand STD initial slope (H6'; 100%)

	STD^{max}	k_{sat}	STD_0	STD (%)
**H1**	22.22	0.49	10.91	87
**H3**	19.49	0.61	11.81	94
**H6'**	10.27	1.22	12.55	100

1.2.5. Glucosamine-1-phosphate (GlcN-1-P)

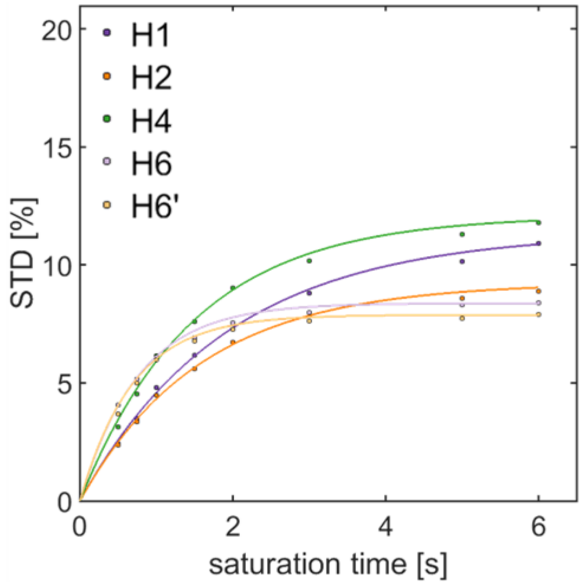

Figure S5: STD build-up curves (symbols: experimental data; solid lines: mathematical fitting to a monoexponential equation) for GlcN-1-P in [D_{11}]Tris buffer, pH 7.4 (NaCl 100 mM) at 278 K. 50 µM binding unit was used for a ligand concentration of 5 mM.

Table S10: STD^{max}, k_{sat} and STD_0 for GlcN-1-P. Relative STD (%) were obtained through normalisation against the largest ligand STD initial slope (H6'; 100%)

	STD^{max}	k_{sat}	STD_0	STD (%)
**H1**	11.39	0.52	5.88	52
**H2**	9.25	0.63	5.83	51
**H4**	12.09	0.67	8.12	71
**H6**	8.38	1.32	11.02	97
**H6'**	7.88	1.44	11.36	100

1.2.6. 6F-Glucose-1-phosphate (6F-Glc-1-P)

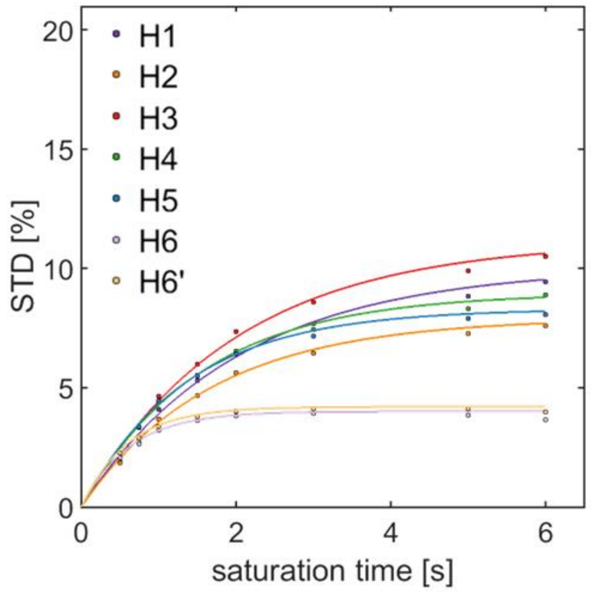

Figure S6: STD build-up curves (symbols: experimental data; solid lines: mathematical fitting to a monoexponential equation) for 6F-Glc-1-P in [D_{11}]Tris buffer, pH 7.4(NaCl 100 mM) at 278 K. 35 µM binding unit was used for a ligand concentration of 3.5 mM.

Table S11: STD^{max}, k_{sat} and STD_0 for 6F-Glc-1-P. Relative STD (%) were obtained through normalisation against the largest ligand STD initial slope (H6'; 100%)

	STD^{max}	k_{sat}	STD_0	STD (%)
**H1**	10.06	0.49	4.94	70
**H2**	7.92	0.60	4.72	67
**H3**	11.16	0.51	5.66	80
**H4**	8.97	0.64	5.78	82
**H5**	8.30	0.74	6.10	86
**H6**	4.01	1.60	6.42	91
**H6'**	4.20	1.69	7.07	100

1.3. Structural basis of molecular recognition of natural and unnatural acceptor-like substrates

Anomeric equilibria and STD NMR intensities. In order to accurately quantitate the STD intensities of ligands with both anomers present in solution, we took into account the different concentrations of α- and β-anomers at equilibrium in solution, and a correction factor was applied to each α- and β- anomer STD intensity. The correction factor was calculated as the ratio of integrals of the α- and β- anomeric protons signals relative to the integral of the H1 signal of the non-reducing ring for D-cellobiose, and the H2 signal of the non-reducing ring for D-cellotriose, and D-laminaribiose. The ratio of integrals between ^{1}H signals of α- and β- anomeric protons were as follows:

	α	β
D-cellobiose	0.15	0.85
D-cellobiose + K_3PO_4 100 µM	0.4	0.6
D-cellobiose in PBS 25 mM	0.3	0.7
D-cellotriose	0.4	0.6
D-laminaribiose	0.45	0.55

1.3.1. STD NMR spectra of CDP-Acceptors binding

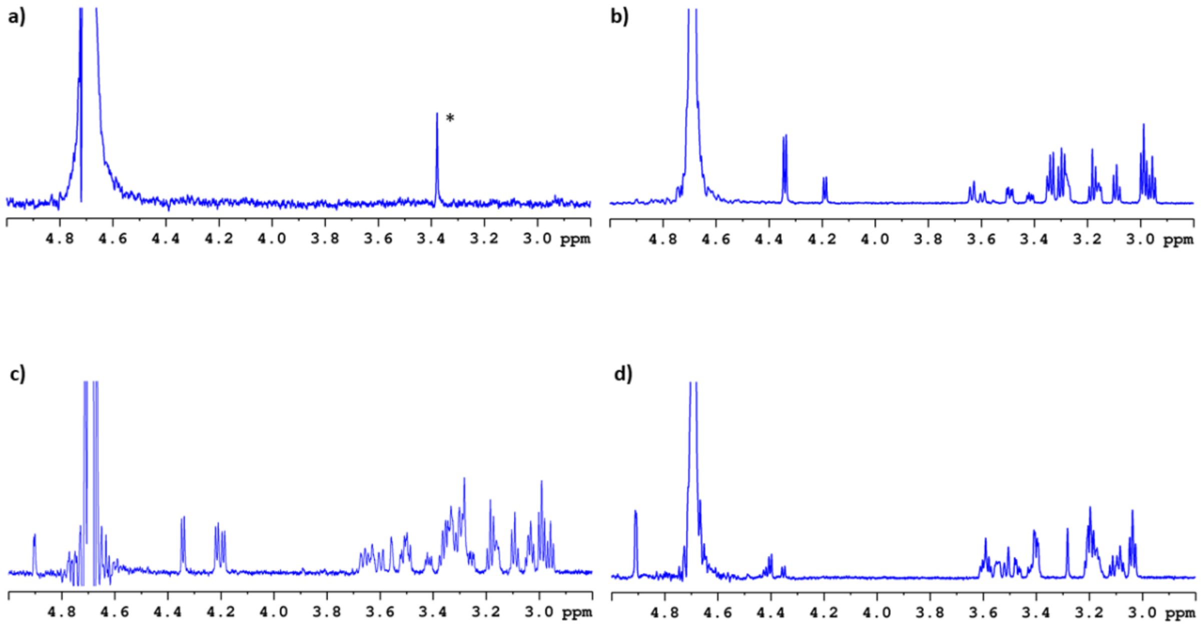

Figure S7: STD NMR difference (off resonance – on resonance) spectra of a) D-glucose, b) D-cellobiose, c) D-cellotriose and d) D-laminaribiose in [D_{11}]Tris buffer 25 mM, pH 7.4 at 6 seconds saturation time. For D-glucose, the protein:ligand ratio for the collected spectra was 1:20. In this case, the STD NMR difference spectrum do not show any peak from the sugar, indicating lack of binding between D-glucose and CDP. The peak labelled with the asterisk in D-glucose spectra belongs to the residual Tris-CH_2 signal and gives a residual STD% of 1.5% at saturation time 6 s.

1.3.2. D-cellobiose

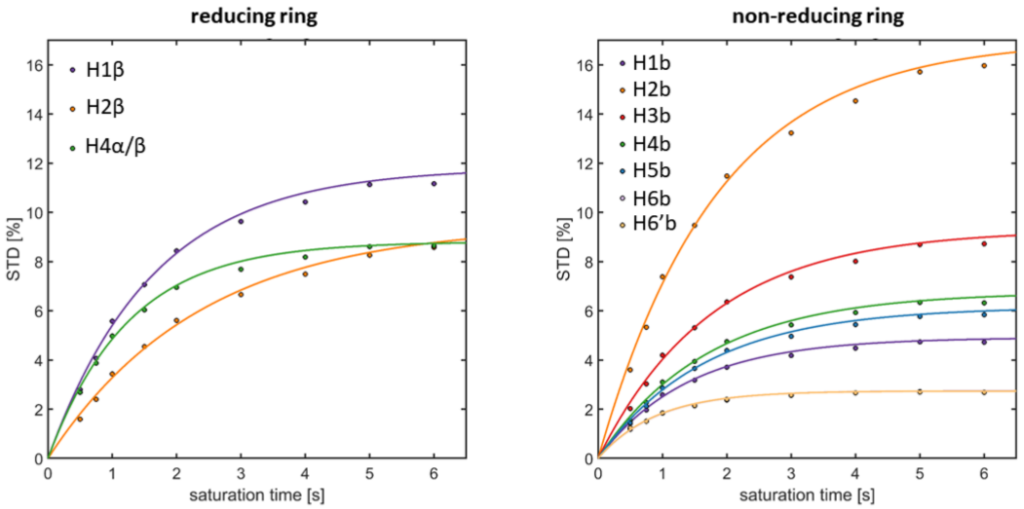

Figure S8: D-cellobiose NMR build-up curves recorded at increasing saturation time (from 0.5 to 6 seconds) in $[D_{11}]$Tris buffer 25 mM, pH 7.4 at 278 K. 15 µM binding unit was used for a ligand concentration of 3 mM.

Table S12: STD^{max}, ksat and STD_0 for D-cellobiose in $[D_{11}]$Tris buffer 25 mM, pH 7.4 The assigned STD % were obtained through normalisation against the maximum ligand STD initial slope (H2 terminal non-reducing ring; 100%)

	STD^{max}	k_{sat}	STD_0	STD (%)
**H1β**	11.82	0.61	7.24	78.52
**H2β**	9.52	0.42	4.02	43.68
**H4α/β**	8.81	0.80	7.05	76.55
**H1b**	4.92	0.71	3.51	38.04
**H2b**	17.03	0.54	9.21	100.00
**H3b**	9.30	0.57	5.28	57.27
**H4b**	6.76	0.59	3.97	43.05
**H5b**	6.16	0.60	3.71	40.24
**H6b**	2.74	1.10	3.01	32.65
**H6'b**	2.74	1.11	3.05	33.05

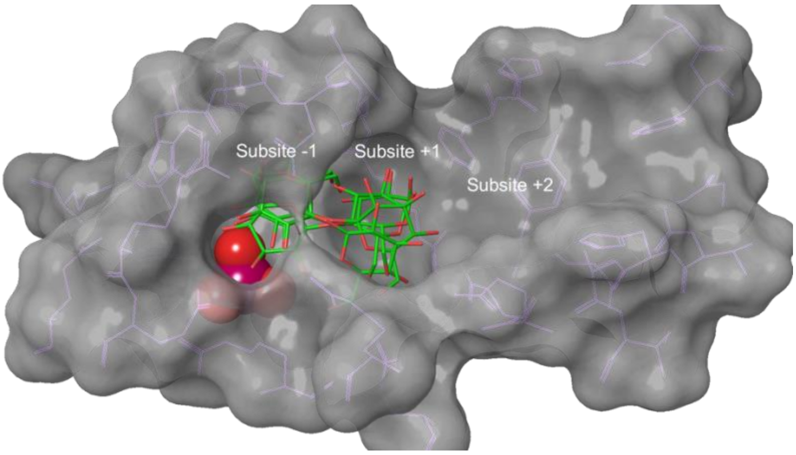

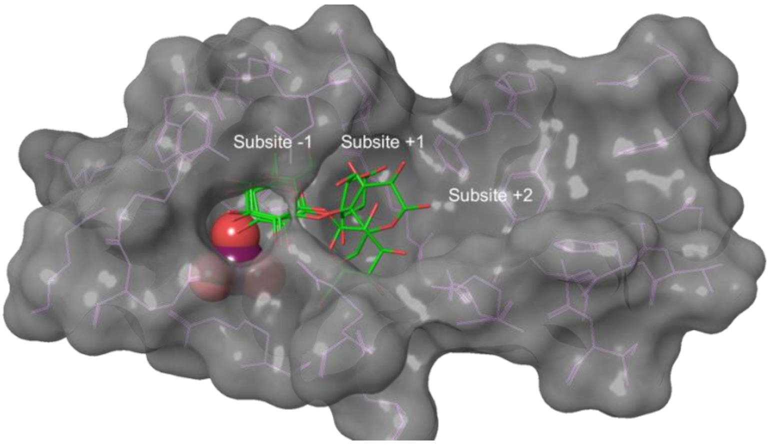

Figure S9: Docking calculations of the complexes of CDP with α-D-cellobiose (top) and β-D-cellobiose (bottom). Both figures show the superposition of the best scored representative within each of the 4 clusters identified for each ligand.

Table S13: Clusters rank and population of CDP complex with D-cellobiose. A total number of 19 poses were obtained for both the α- and β-anomers. The computed binding energy (Glide emodel) and the RMSD refer to the most energetically favourable pose obtained for each cluster.

**D-cellobiose α-anomer**				**D-cellobiose β-anomer**			
**Cluster rank**	**Cluster population (%)**	**Glide emodel (kcal/mol)**	**RMSD (Å)**	**Cluster rank**	**Cluster population (%)**	**Glide emodel (kcal/mol)**	**RMSD (Å)**
**2**	79	-80.26	1.33	**4**	42	-77.53	0.93
**1**	11	-69.34	0.53	**3**	32	-74.79	0.85
**3**	5	-65.64	--[(a)]	**2**	21	-70.12	0.85
**4**	5	-66.82	--	**1**	5	-58.73	--

(a) Cells with “--" indicate clusters with only one molecule, so that no value of RMSD is given

Table S14: ϕ and ψ torsional angles for D-cellobiose anomers, indicating that the two ligands have a different inter-glycosidic conformation, as reported by the different ψ angles. Nonetheless, both the conformation were in the allowed region of the energetic map, as reported by GlycoMapsDB (Glycosciences.de).[32] ϕ and ψ are defined following NMR spectroscopy criteria as $\Phi \equiv H_1 - C_1 - O_g - C_x$ and $\Psi \equiv C_1 - O_g - C_x - H_x$ (where H_1 is the hydrogen attached to the anomeric carbon, C_1 is the anomeric carbon, O_g is the glycosidic oxygen and C_x is the carbon belonging to the following residue, linked to the glycosidic oxygen).

	ϕ	ψ
**α-anomer**	44.5	-27.8
**β-anomer**	45.3	-3.7

1.3.3. D-cellotriose

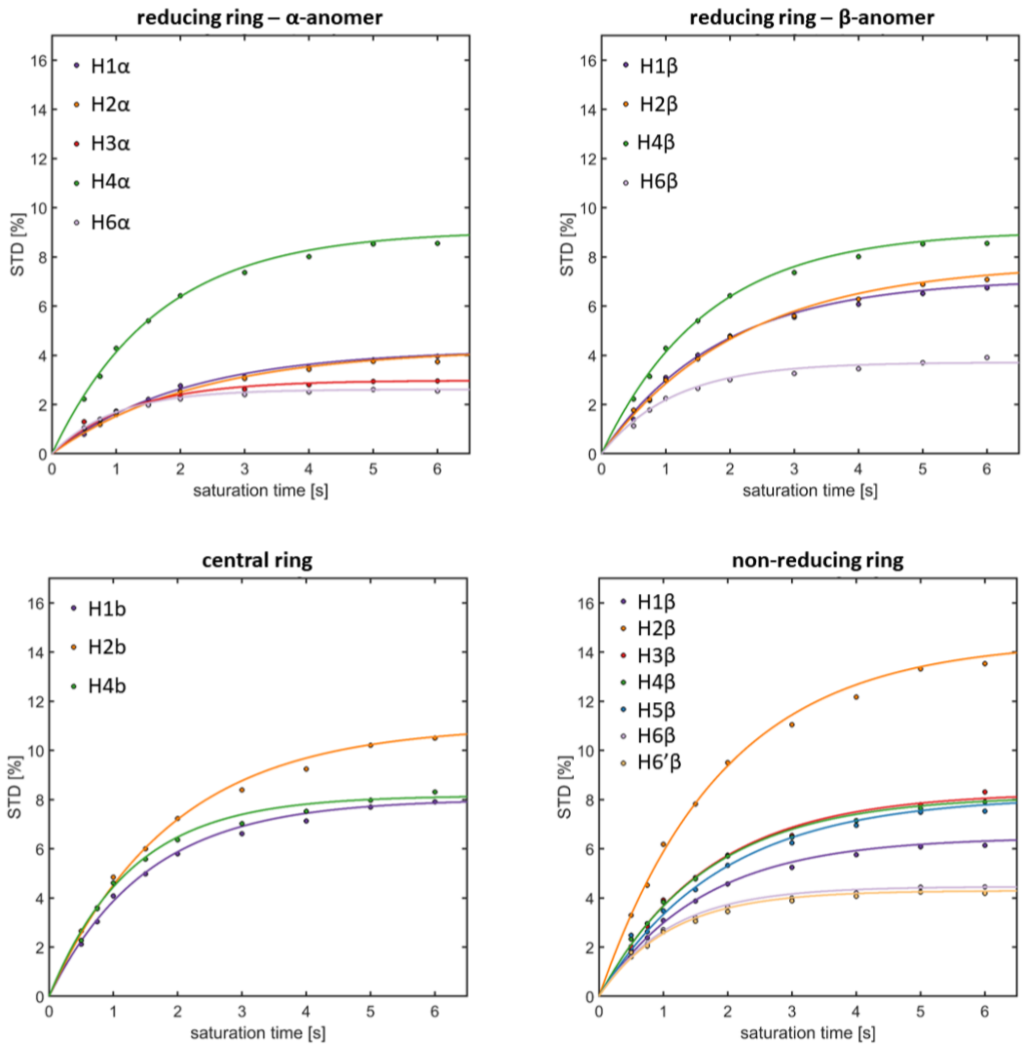

Figure S10: D-cellotriose NMR build-up curves recorded at increasing saturation time (from 0.5 to 6 seconds) in [D11]Tris buffer 25 mM, pH 7.4 at 278 K. 15 µM binding unit was used for a ligand concentration of 3 mM.

Table S15: STD^{max}, ksat and STD_0 for D-cellobiose in [D_{11}]Tris buffer 25 mM, pH 7.4. The assigned STD % were obtained through normalisation against the maximum ligand STD initial slope (H2 terminal non-reducing ring; 100%)

	STD^{max}	k_{sat}	STD_0	STD (%)
**H1α**	4.24	0.49	2.06	27.25
**H2α**	4.26	0.44	1.88	24.93
**H3α**	2.98	0.82	2.44	32.33
**H4α**	9.06	0.61	5.52	73.09
**H6α**	2.61	1.04	2.71	35.81
**H1ß**	7.11	0.54	3.86	51.13
**H2ß**	7.75	0.46	3.55	47.03
**H4ß**	9.06	0.61	5.52	73.09
**H6ß**	3.71	0.89	3.30	43.66
**H1b**	8.02	0.66	5.29	70.01
**H2b**	11.01	0.53	5.84	77.25
**H4b**	8.16	0.79	6.43	85.14
**H1c**	6.47	0.62	4.00	52.99
**H2c**	14.46	0.52	7.56	100.00
**H3c**	8.29	0.59	4.87	64.44
**H4c**	8.15	0.60	4.87	64.42
**H5c**	8.13	0.53	4.29	56.71
**H6c**	4.46	0.90	4.03	53.30
**H6'c**	4.30	0.90	3.87	51.21

1.3.4. D-laminaribiose

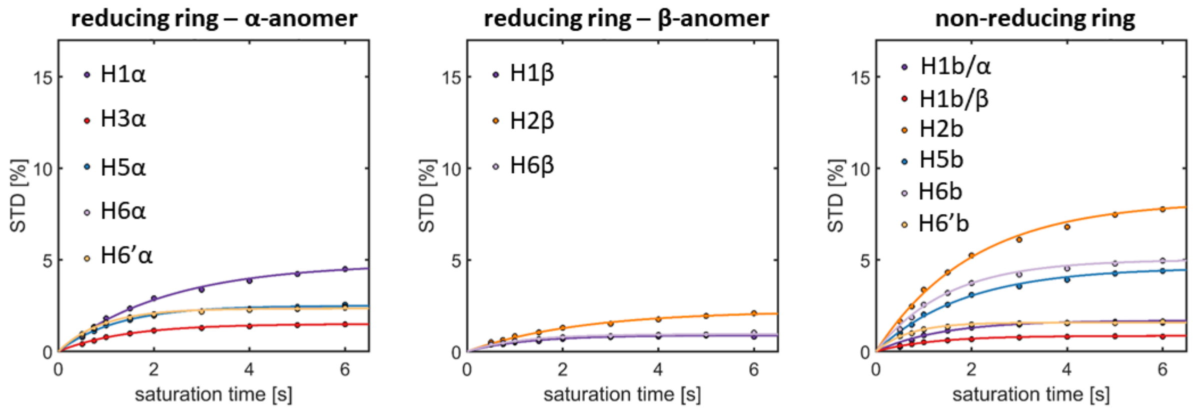

Figure S11: D-laminaribiose NMR build-up curves recorded at increasing saturation time (from 0.5 to 6 seconds) in [D_{11}]Tris buffer 25 mM, pH 7.4 at 278 K. 10 µM binding unit was used for a ligand concentration of 2 mM.

Table S16: STD^{max}, ksat and STD_0 for D-cellotriosyl-azide in [D_{11}]Tris buffer 25 mM, pH 7.4 . The assigned STD % were obtained through normalisation against the maximum ligand STD initial slope (H2 terminal non-reducing ring; 100%)

	STD^{max}	k_{sat}	STD_0	STD (%)
**H1α**	5.41	0.44	2.38	58.87
**H3α**	1.69	0.71	1.20	29.58
**H5α**	2.81	0.80	2.25	55.66
**H6α**	2.65	1.08	2.87	70.85
**H6'α**	2.64	1.10	2.90	71.49
**H1β**	0.81	0.79	0.64	15.89
**H2β**	2.03	0.43	0.87	21.38
**H6β**	0.87	0.90	0.79	19.41
**H6β**	0.99	1.08	1.07	26.49
**H1b/α**	1.86	0.72	1.34	33.03
**H1b/β**	0.78	0.85	0.66	16.34
**H2b**	8.23	0.49	4.05	100.00
**H5b**	4.59	0.54	2.50	61.67
**H6b**	5.02	0.68	3.40	83.98
**H6'b**	1.58	1.44	2.28	56.28

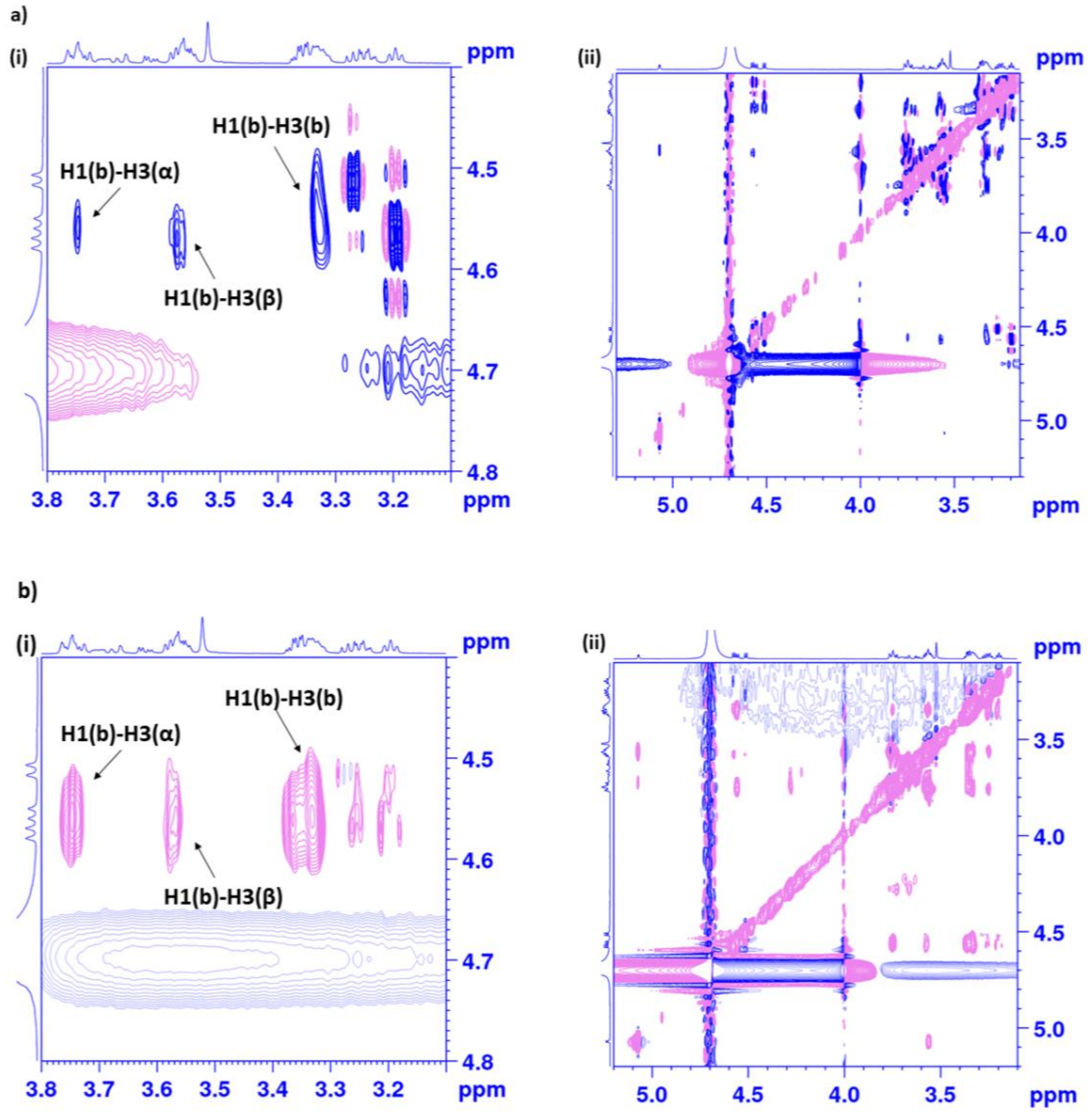

Figure S12: (i) Expansion and (ii) full spectra of the transferred 2D-NOESY spectra of a) free D-laminaribiose and b) D-laminaribiose bound to CDP (1:10 protein to ligand ratio) in [D_{11}]Tris buffer 25 mM pH 7.4, NaCl 100 mM registered at 300 ms mixing time and 290 K in a 800 MHz spectrometer.

1.4. Impact of phosphate on acceptor binding

1.4.1. Determination of acceptor binding epitope upon phosphate titration

<u>**CDP/D-cellobiose complex with small excess of inorganic phosphate**</u>

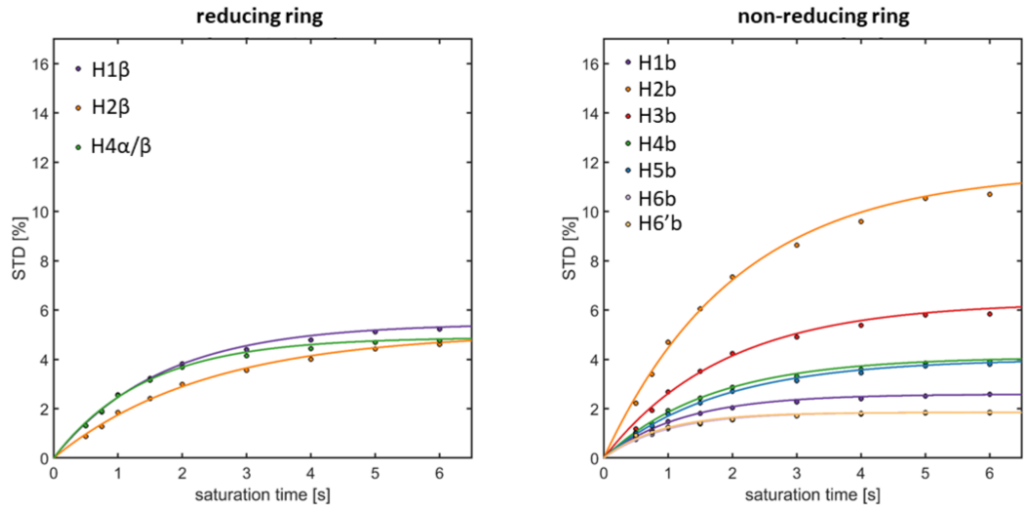

Figure S13: D-cellobiose NMR build-up curves recorded at increasing saturation time (from 0.5 to 6 seconds) in [D_{11}]Tris buffer 25 mM, pH 7.4 at 278 K. 15 µM binding unit was used for a ligand concentration of 3 mM and a inorganic phosphate concentration of 100 µM.

Table S17: STD^{max}, ksat and STD_0 for D-cellobiose in [D_{11}]Tris buffer 25 mM, pH 7.4 with K_3PO_4 100 µM. The assigned STD % were obtained through normalisation against the maximum ligand STD initial slope (H2 terminal non-reducing ring; 100%)

	STD^{max}	k_{sat}	STD_0	STD (%)
**H1β**	5.46	0.60	3.27	58.09
**H2β**	5.09	0.42	2.14	37.98
**H4α/β**	4.90	0.70	3.43	60.85
**H1b**	2.59	0.82	2.11	37.44
**H2b**	11.65	0.48	5.63	100.00
**H3b**	6.32	0.53	3.37	59.89
**H4b**	4.10	0.60	2.47	43.77
**H5b**	4.02	0.55	2.21	39.16
**H6b**	1.85	1.00	1.86	33.05
**H6'b**	1.85	1.10	2.04	36.19

CDP/D-cellobiose complex with large excess of inorganic phosphate

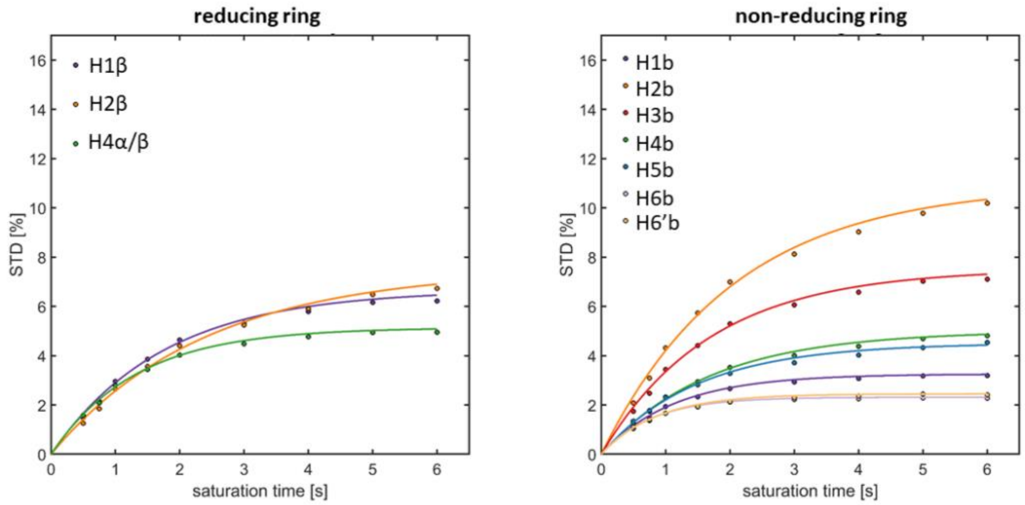

Figure S14: D-cellobiose NMR build-up curves recorded at increasing saturation time (from 0.5 to 6 seconds) in PBS 25 mM, pH 7.4 at 278 K. 15 µM binding unit was used for a ligand concentration of 3 mM.

Table S18: STD^{max}, ksat and STD_0 for D-cellobiose in PBS 25 mM, pH 7.4. The assigned STD % were obtained through normalisation against the maximum ligand STD initial slope (H2 terminal non-reducing ring; 100%)

	STD^{max}	k_{sat}	STD_0	STD (%)
**H1β**	6.67	0.57	3.81	71.80
**H2β**	7.51	0.42	3.14	59.18
**H4α/β**	5.14	0.76	3.90	73.33
**H1b**	3.26	0.88	2.87	53.95
**H2b**	10.94	0.49	5.31	100.00
**H3b**	7.52	0.59	4.43	83.47
**H4b**	4.85	0.59	2.86	53.76
**H5b**	4.51	0.67	3.03	57.00
**H6b**	2.31	1.28	2.97	55.95
**H6'b**	2.45	1.16	2.83	53.37

Table S19: Average STD intensities reduction for D-cellobiose in [D_{11}]Tris buffer 25 mM, pH 7.4 at 278 K with a inorganic phosphate concentration of 100 µM and in PBS 25 mM, pH 7.4 at 278 K, in comparison with the STD intensities recorded in [D_{11}]Tris buffer 25 mM, pH 7.4 at 278 K. The percentage of STD signal reduction was calculated as a ratio between the difference of the STD intensities recorded in the absence and in the presence of inorganic phosphate and the STD intensities recorded in the absence of inorganic phosphate. Then, the average value was measured.

	[D_{11}]Tris buffer + 100 µM K_3PO_4	*PBS 25 mM*
**H1β**	38.7 %	45.6 %
**H2β**	28.8 %	21.7 %
**H4β**	50.8 %	53.2 %
**H1b**	44.5 %	27.8 %
**H2b**	35.3 %	39.6 %
**H3b**	34.8 %	17.8 %
**H4b**	38.0 %	24.6 %
**H5b**	36.8 %	21.0 %
**H6b**	33.9 %	12.0 %
average	38.0 %	29.3 %

1.4.2. Determination of D-cellobiose bioactive conformation upon phosphate titration

tr-NOESY experiments are important for the investigation of ^{1}H-^{1}H distances and conformational rearrangement upon ligand binding

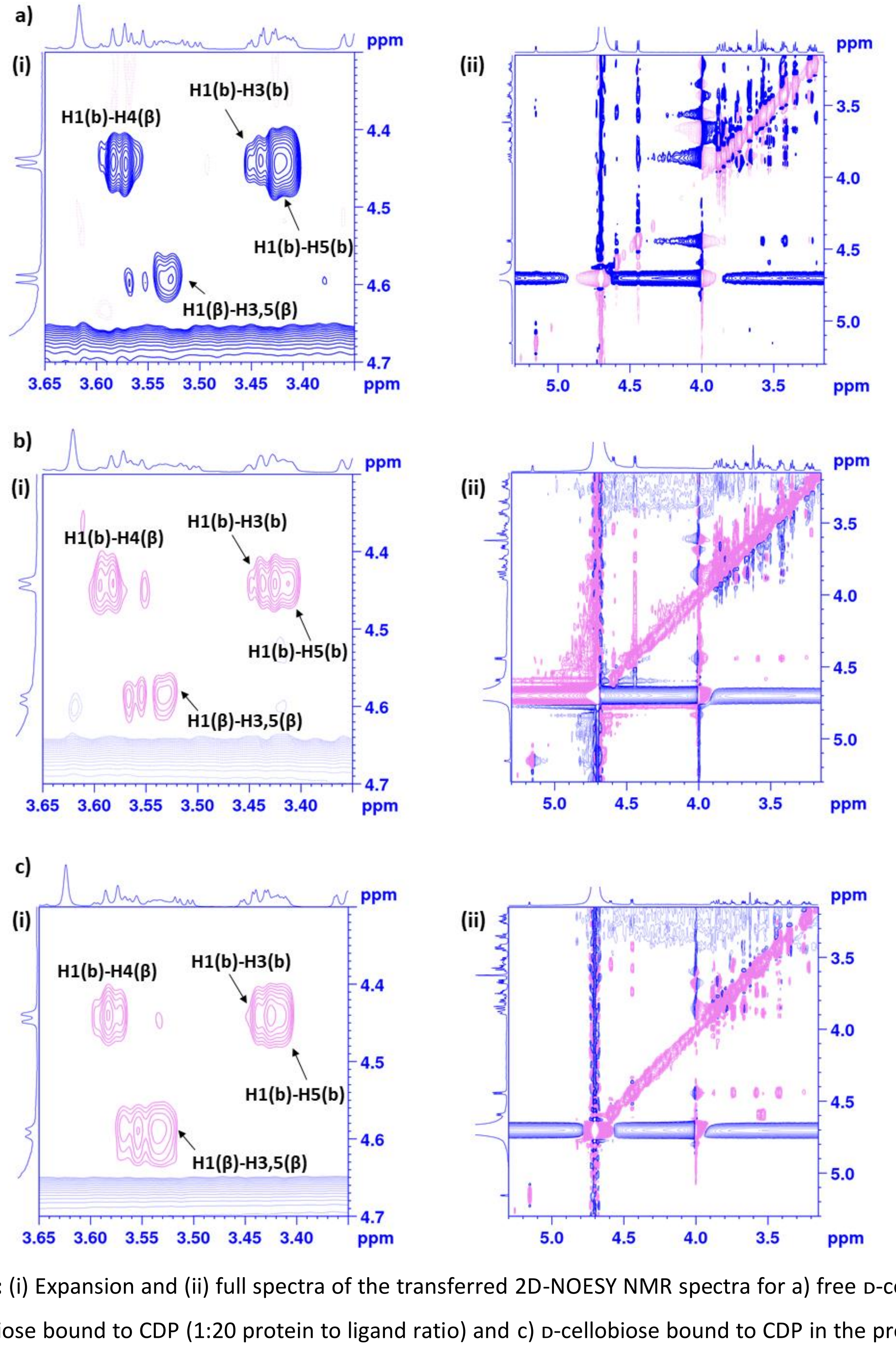

Figure S15: (i) Expansion and (ii) full spectra of the transferred 2D-NOESY NMR spectra for a) free D-cellobiose, b) D-cellobiose bound to CDP (1:20 protein to ligand ratio) and c) D-cellobiose bound to CDP in the presence of inorganic phosphate in [D_{11}]Tris buffer 25 mM, pH 7.4 NaCl 100 mM recorded at 160 ms mixing time and 298 K in a 800 MHz spectrometer.

Table S20: ^{1}H-^{1}H distances (Å) measured via tr-NOESY experiments for D-cellobiose as free ligand, when bound to CDP (20:1 ligand:enzyme ratio) and bound to CDP in the presence of 5-fold excess per binding site of phosphate.

	Free D-cellobiose		Bound D-cellobiose		Bound D-cellobiose (phosphate +)	
Proton pairs	*distance (Å)*	*σ*	*distance (Å)*	*σ*	*distance (Å)*	*σ*
**H1(b)-H5(b)**	2.38	0.03	2.38	-0.10	2.38	-0.04
**H1(b)-H3(b)**	3.07	0.01	2.91	-0.03	2.83	-0.01
**H1(b)-H4(β)**	2.54	0.02	2.44	-0.09	2.38	-0.04
**H4(b)-H6(b)**	2.92	0.01	3.11	-0.02	n.r	n.r
**H4(b)-H6'(b)**	3.53	0.003	3.06	-0.02	2.95	-0.01
**H2β-H4β**	2.52	0.02	2.21	-0.16	2.05	-0.09
**H1(b)-H6(β)**	n.r.	n.r.	2.46	-0.08	2.66	-0.02
**H1(b)-H6'(β)**	n.r.	n.r,	2.62	-0.06	2.46	-0.03

1.4.3. Structural details of the interaction in the CDP/Glc-1-P/D-cellobiose ternary complex

Table S21: Clusters rank and population of CDP/Glc-1-P/ D-cellobiose ternary complex. A total number of 1363 poses were obtained. The computed binding energy (Glide emodel) refer to the most energetically favourable pose obtained for each cluster and the RMSD to the average root-mean-square-deviation within the cluster.

Cluster rank	Cluster population (%)	Glide emodel (kcal/mol)	RMSD (Å)
1	79.6%	-62.63	0.57
2	9.2%	-59.27	0.52
3	5.2%	-54.51	0.56
4	3.3%	-57.38	0.62
5	2.7%	-42.36	0.62

References

1. Nakai, H.; Hachem, M. A.; Petersen, B. O.; Westphal, Y.; Mannerstedt, K.; Baumann, M. J.; Dilokpimol, A.; Schols, H. A.; Duus, J. Ø.; Svensson, B., Efficient chemoenzymatic oligosaccharide synthesis by reverse phosphorolysis using cellobiose phosphorylase and cellodextrin phosphorylase from Clostridium thermocellum. *Biochimie* **2010,** *92* (12), 1818-1826.
2. Shintate, K.; Kitaoka, M.; Kim, Y.-K.; Hayashi, K., Enzymatic synthesis of a library of β-(1→4) hetero- d-glucose and d-xylose-based oligosaccharides employing cellodextrin phosphorylase. *Carbohydrate Research* **2003,** *338* (19), 1981-1990.
3. O'Neill, E. C.; Pergolizzi, G.; Stevenson, C. E. M.; Lawson, D. M.; Nepogodiev, S. A.; Field, R. A., Cellodextrin phosphorylase from Ruminiclostridium thermocellum: X-ray crystal structure and substrate specificity analysis. *Carbohydrate Research* **2017,** *451*, 118-132.
4. Singh, R. P.; Pergolizzi, G.; Nepogodiev, S. A.; de Andrade, P.; Kuhaudomlarp, S.; Field, R. A., Preparative and Kinetic Analysis of beta-1,4- and beta-1,3-Glucan Phosphorylases Informs Access to Human Milk Oligosaccharide Fragments and Analogues Thereof. *Chembiochem* **2020,** *21* (7), 1043-1049.
5. De Andrade, P.; Muñoz-García, J. C.; Pergolizzi, G.; Gabrielli, V.; Nepogodiev, S. A.; Iuga, D.; Fábián, L.; Nigmatullin, R.; Johns, M. A.; Harniman, R.; Eichhorn, S. J.; Angulo, J.; Khimyak, Y. Z.; Field, R. A., Chemoenzymatic synthesis of fluorinated cellodextrins identifies a new allomorph for cellulose-like materials. *Chemistry – A European Journal* **2020**.
6. Samain, E.; Lancelon-Pin, C.; Férigo, F.; Moreau, V. S., 1995 #25}; Chanzy, H.; Heyraud, A.; Driguez, H., Phosphorolytic synthesis of cellodextrins. **1995,** *271* (2), 217-226.
7. Hiraishi, M.; Igarashi, K.; Kimura, S.; Wada, M.; Kitaoka, M.; Samejima, M., Synthesis of highly ordered cellulose II in vitro using cellodextrin phosphorylase. *Carbohydrate Research* **2009,** *344* (18), 2468-2473.
8. Yataka, Y.; Sawada, T.; Serizawa, T., Enzymatic synthesis and post-functionalization of two-dimensional crystalline cellulose oligomers with surface-reactive groups. **2015,** *51* (63), 12525-12528.
9. Serizawa, T.; Kato, M.; Okura, H.; Sawada, T.; Wada, M., Hydrolytic activities of artificial nanocellulose synthesized via phosphorylase-catalyzed enzymatic reactions. *Polymer Journal* **2016,** *48* (4), 539-544.
10. Hata, Y.; Kojima, T.; Koizumi, T.; Okura, H.; Sakai, T.; Sawada, T.; Serizawa, T., Enzymatic Synthesis of Cellulose Oligomer Hydrogels Composed of Crystalline Nanoribbon Networks under Macromolecular Crowding Conditions. **2017,** *6* (2), 165-170.
11. Hata, Y.; Sawada, T.; Serizawa, T., Effect of solution viscosity on the production of nanoribbon network hydrogels composed of enzymatically synthesized cellulose oligomers under macromolecular crowding conditions. *Polymer Journal* **2017,** *49* (7), 575-581.
12. Serizawa, T.; Fukaya, Y.; Sawada, T., Self-Assembly of Cellulose Oligomers into Nanoribbon Network Structures Based on Kinetic Control of Enzymatic Oligomerization. *Langmuir* **2017,** *33* (46), 13415-13422.
13. Hata, Y.; Fukaya, Y.; Sawada, T.; Nishiura, M.; Serizawa, T., Biocatalytic oligomerization-induced self-assembly of crystalline cellulose oligomers into nanoribbon networks assisted by organic solvents. *Beilstein Journal of Nanotechnology* **2019,** *10*, 1778-1788.
14. Hata, Y.; Sawada, T.; Marubayashi, H.; Nojima, S.; Serizawa, T., Temperature-Directed Assembly of Crystalline Cellulose Oligomers into Kinetically Trapped Structures during Biocatalytic Synthesis. *Langmuir* **2019,** *35* (21), 7026-7034.
15. Zhang, Y. H. P.; Lynd, L. R., Biosynthesis of radiolabeled cellodextrins by the Clostridium thermocellum cellobiose and cellodextrin phosphorylases for measurement of intracellular sugars. **2006,** *70* (1), 123-129.
16. Zhong, C.; Zajki-Zechmeister, K.; Nidetzky, B., Reducing end thiol-modified nanocellulose: Bottom-up enzymatic synthesis and use for templated assembly of silver nanoparticles into biocidal composite material. *Carbohydrate Polymers* **2021,** *260*, 117772.

17. Hanamura, M.; Sawada, T.; Serizawa, T., In-Paper Self-Assembly of Cellulose Oligomers for the Preparation of All-Cellulose Functional Paper. *ACS Sustainable Chemistry & Engineering* **2021,** *9* (16), 5684-5692.
18. Nohara, T.; Sawada, T.; Tanaka, H.; Serizawa, T., Enzymatic Synthesis of Oligo(ethylene glycol)-Bearing Cellulose Oligomers for in Situ Formation of Hydrogels with Crystalline Nanoribbon Network Structures. **2016**.
19. Yataka, Y.; Sawada, T.; Serizawa, T., Multidimensional Self-Assembled Structures of Alkylated Cellulose Oligomers Synthesized via in Vitro Enzymatic Reactions. **2016,** *32* (39), 10120-10125.
20. Nohara, T.; Sawada, T.; Tanaka, H.; Serizawa, T., Enzymatic synthesis and protein adsorption properties of crystalline nanoribbons composed of cellulose oligomer derivatives with primary amino groups. *J Biomater Sci Polym Ed* **2017,** *28* (10-12), 925-938.
21. Wang, J.; Niu, J.; Sawada, T.; Shao, Z.; Serizawa, T., A Bottom-Up Synthesis of Vinyl-Cellulose Nanosheets and Their Nanocomposite Hydrogels with Enhanced Strength. *Biomacromolecules* **2017,** *18* (12), 4196-4205.
22. Adharis, A.; Petrović, D. M.; Özdamar, I.; Woortman, A. J. J.; Loos, K., Environmentally friendly pathways towards the synthesis of vinyl-based oligocelluloses. *Carbohydrate Polymers* **2018,** *193*, 196-204.
23. Krishnareddy, M.; Kim, Y.-K.; Kitaoka, M.; Mori, Y.; Hayashi, K., Cellodextrin Phosphorylase from Clostridium thermocellum YM4 Strain Expressed in Escherichia coli. **2002,** *49* (1), 1-8.
24. Serizawa, T.; Maeda, T.; Sawada, T., Neutralization-Induced Self-Assembly of Cellulose Oligomers into Antibiofouling Crystalline Nanoribbon Networks in Complex Mixtures. *ACS Macro Letters* **2020**, 301-305.
25. Petrović, D. M.; Kok, I.; Woortman, A. J. J.; Ćirić, J.; Loos, K., Characterization of Oligocellulose Synthesized by Reverse Phosphorolysis Using Different Cellodextrin Phosphorylases. *Analytical Chemistry* **2015,** *87* (19), 9639-9646.
26. Moreau, V.; Viladot, J. L.; Samain, E.; Planas, A.; Driguez, H., Design and chemoenzymatic synthesis of thiooligosaccharide inhibitors of 1,3:1,4-beta-D-glucanases. *Bioorg Med Chem* **1996,** *4* (11), 1849-55.
27. Ambar; Kitaoka, M.; Hayashi, K., Synthesis of a Cellobiosylated Dimer and Trimer and of Cellobiose-Coated Polyamidoamine (PAMAM) Dendrimers to Study Accessibility of an Enzyme, Cellodextrin Phosphorylase. **2003,** *2003* (13), 2462-2470.
28. Alexander, K. S. A. J. K., Purification and Properties of β-1,4-Oligoglucan:Orthophosphate Glucosyltransferase from Clostridium thermocellum. *Journal Biological Chemistry* **1969,** *244*, 457-464.
29. Kawaguchi, T.; Ikeuchi, Y.; Tsutsumi, N.; Kan, A.; Sumitani, J.-I.; Arai, M., Cloning, nucleotide sequence, and expression of the Clostridium thermocellum cellodextrin phosphorylase gene and its application to synthesis of cellulase inhibitors. **1998,** *85* (2), 144-149.
30. Pergolizzi, G.; Kuhaudomlarp, S.; Kalita, E.; Field, R. A., Glycan Phosphorylases in Multi-Enzyme Synthetic Processes. *Protein Pept Lett* **2017,** *24* (8), 696-709.
31. Sheth, K.; Alexander, J., Cellodextrin phosphorylase from Clostridium thermocellum. *Biochimica et Biophysica Acta (BBA) - General Subjects* **1967,** *148* (3), 808-810.
32. Böhm, M.; Bohne-Lang, A.; Frank, M.; Loss, A.; Rojas-Macias, M. A.; Lütteke, T., Glycosciences.DB: an annotated data collection linking glycomics and proteomics data (2018 update). *Nucleic Acids Research* **2019,** *47* (D1), D1195-D1201.
